# MXenes for Infrared Thermal Management

**DOI:** 10.1021/acsnano.5c14464

**Published:** 2025-11-28

**Authors:** Tufail Hassan, Changhoon Park, Shabbir Madad Naqvi, Jongyoun Kim, Hyunho Kim, Zubair Khalid, Sungmin Jung, Yury Gogotsi, Chong Min Koo

**Affiliations:** † School of Advanced Materials Science and Engineering, 35017 Sungkyunkwan University, Seobu-ro 2066, Jangan-gu, Suwon-si, Gyeonggi-do 16419, Republic of Korea; ‡ Materials Science and Engineering Department and A. J. Drexel Nanomaterials Institute, 6527 Drexel University, 3141 Chestnut Street, Philadelphia, Pennsylvania 19104, United States; § School of Chemical Engineering, Sungkyunkwan University, Seobu-ro 2066, Jangan-gu, Suwon-si, Gyeonggi-do 16419, Republic of Korea

**Keywords:** MXenes, infrared radiation, optical properties, thermal management, camouflage, photothermal, electrothermal, spectral selectivity, anticounterfeiting, thermal therapy

## Abstract

Two-dimensional (2D) MXenes have recently emerged as a distinctive class of materials for advanced thermal management due to their combination of high electrical conductivity, broadband optical absorption, low or high infrared (IR) emissivity, efficient light-to-heat conversion, and anisotropic thermal conductivity. This review provides a thorough overview of MXene interaction with IR radiation and MXene-based thermal management strategies, emphasizing the fundamental mechanisms governing spectral response, heat generation, transfer, and radiative emission. We review recent progress in the development of MXene films, coatings, aerogels, fibers, and composites for applications such as ultrathin thermal insulation, IR camouflage, photothermal therapy, wound healing, solar water desalination, wearable heaters, deicing systems, soft actuators, and separation membranes. Finally, we address prevailing challenges and future research directions, offering perspectives to facilitate the advancement of next-generation MXene-based materials for advanced thermal management.

## Graphical abstract

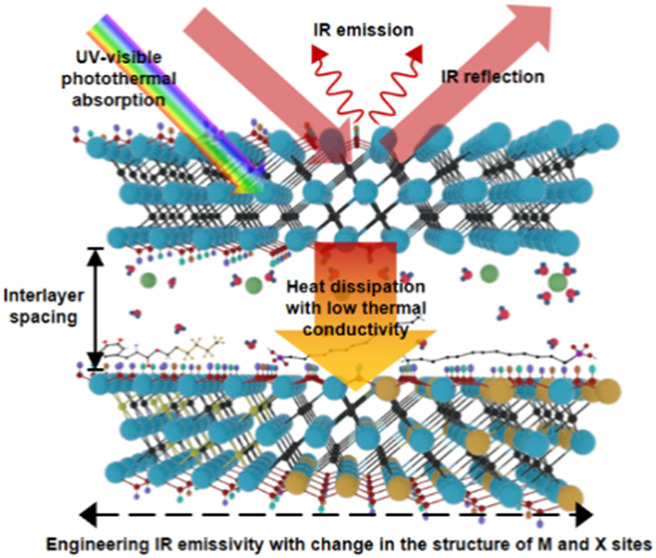

## 1 Introduction

Efficient thermal management is crucial for ensuring performance, safety, and energy efficiency across a wide range of advanced technologies, including electronics, mobility systems, aerospace, water treatment, healthcare, textiles, and zero-energy buildings. In particular, applications such as heat dissipation, infrared (IR) shielding, thermal camouflage, and adaptive heating demand materials capable of simultaneously regulating heat generation, transport, and radiative emission.^1,2^ However, conventional thermal management materials, including metals and carbon-based structures, face inherent limitations, such as poor spectral selectivity, high out-of-plane thermal conductivity, and limited tunability of their optical, thermal, and electrical properties.^3–6^ These constraints underscore the urgent need for multifunctional, tailorable materials for the next generation of thermal regulation.

MXenes, a family of two-dimensional (2D) transition metal carbides and nitrides discovered in 2011, have emerged as promising candidates to address these challenges.^7^ With the general formula M_*n*+1_X*_n_* T_*x*_—where M denotes a transition metal (e.g., Ti, V, Nb, Mo), X represents carbon and/or nitrogen, and T_*x*_ refers to surface terminations such as −O, −OH, halogens, chalcogens, etc. MXenes combine metallic electrical conductivity, strong broadband light absorption, low IR emissivity, and significant thermal anisotropy, making them promising for advanced thermal management applications such as thermal camouflage, photothermal conversion, and joule heating.^8,9^ Figure 1 presents a chronological timeline summarizing the key advancements in MXene-based thermal management and related applications from 2017 to 2023.

**Figure 1:**
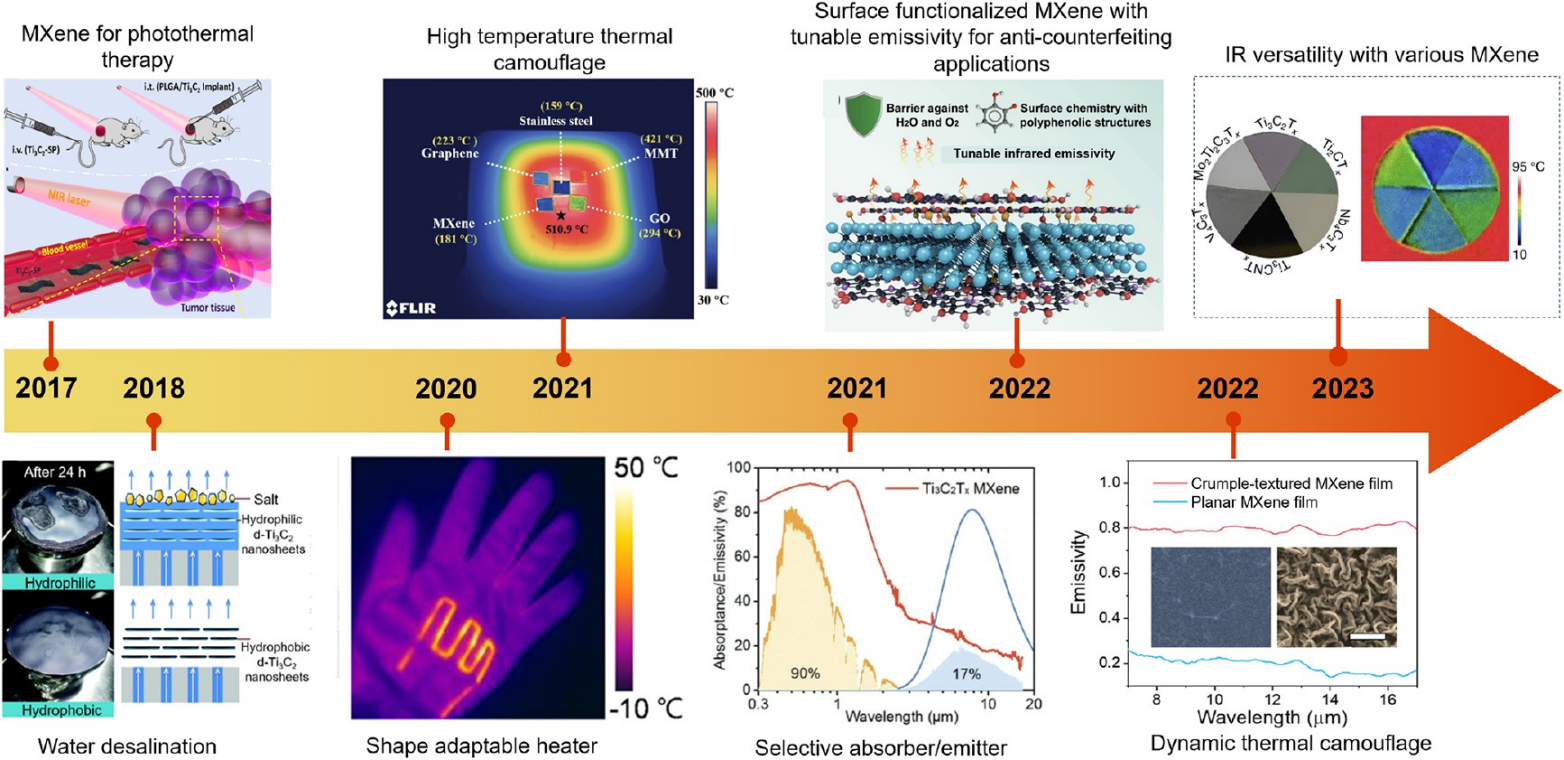
Chronological timeline illustrating the progressive development of MXene-based thermal management and its emerging applications. Since 2017, when the efficient photothermal conversion capability of MXene was reported, they have been studied for use in photothermal therapy, solar-driven water desalination, and biomedical imaging. In 2020, the first shape-adaptable and transparent MXene heaters were introduced, showcasing outstanding Joule heating performance. By 2021, Ti_3_C_2_T_*x*_ MXene demonstrated low thermal emissivity and spectrally selective infrared regulation, attracting considerable attention and suppressed out-of-plane thermal conductivity. Subsequent advancements through surface functionalization, dynamic camouflage design, and compositional tailoring across various transition-metal MXenes have further enabled precise control over infrared emissivity and heat-management characteristics. Reproduced with permission.^6,10,19,27–31^ Copyright 2021, John Wiley and Sons; Copyright 2023, Elsevier; Copyright 2019, American Chemical Society; Copyright 2017, American Chemical Society; Copyright 2018, Royal Society of Chemistry; Copyright 2021, John Wiley and Sons; Copyright 2022, American Chemical Society; and Copyright 2022, John Wiley and Sons, respectively.

For instance, Ti_3_C_2_T_*x*_ MXene demonstrates broadband optical absorption across the ultraviolet to mid-infrared spectrum (up to ∼90% in the solar range)^6^ and exhibits a particularly low IR emissivity (∼0.047 in the 3–25 μm band),^10^ providing superior spectral selectivity compared to conventional black absorbers. Regarding thermal transport, Ti_3_C_2_T_*x*_ MXene exhibits high in-plane thermal conductivity (∼55 W m^–1^ K^–1^)^11^ and low out-of-plane thermal conductivity (∼1.78 W m^–1^ K^–1^ or lower)^12^ as well as low infrared emissivity,^10^ facilitating efficient lateral heat dissipation while minimizing radiative and conductive heat loss. These characteristics establish MXenes as excellent candidates for use in photothermal conversion (e.g., solar-driven desalination or photothermal therapy)^13–15^ and thermal camouflage applications where suppression of thermal signatures is essential.^16,17^

In addition, MXenes offer outstanding electrical conductivity (∼20,000 S cm^–1^ for Ti_3_C_2_T_*x*_),^18^ facilitating rapid, energy-efficient Joule heating in applications such as wearable heaters, deicing, and thermal actuators.^19–21^ Crucially, these attributes can be finely tuned. By selecting different transition metals (M = Ti, V, Nb, Mo, etc.) or synthesizing multielement solid solutions, researchers can regulate charge carrier density and mobility, phonon transport, and light–matter interactions. Surface terminations (−OH, −O, −F, −Cl, etc.) further influence the electronic structure, density of states, and electron–phonon coupling, enabling adjustment of optical, thermal, electrical, and IR emissivity characteristics.^9,22^

In addition to their inherent characteristics, MXenes exhibit excellent solution processability, supporting easy manufacturing of inks, thin films, coatings, fibers, and porous aerogels.^23–26^ This enables 2D MXene flakes to be incorporated into composites and hybrid systems, expanding their roles in advanced thermal management applications. Although significant advances have been achieved in the past 3–4 years, there is a lack of a comprehensive review linking the composition, surface chemistry, and structural features of MXenes with their multifunctional thermal management performance. In particular, the literature has yet to provide a cohesive summary of MXene roles in photothermal conversion, thermal camouflage, Joule heating, and adaptive thermal regulation.

This forward-looking review addresses these knowledge gaps by presenting an in-depth analysis of MXene-based technologies for thermal management. We begin by examining the fundamental principles that determine the electrical, thermal, and optical properties of MXenes, with particular attention to the influence of composition and surface chemistry. Subsequently, we examine recent progress in three key application areas: thermal camouflage/infrared shielding, photothermal energy conversion, and electrothermal Joule heating. Within thermal camouflage, MXenes with intrinsically low infrared emissivity and anisotropic thermal transport characteristics are employed, which facilitate adaptive camouflage and suppression of thermal signatures. For photothermal conversion, the broadband light absorption and low or high thermal radiation properties of MXenes are utilized in applications including solar-driven water desalination, photothermal therapy, and biomedical heating technologies (wound healing, etc.). In the context of electrothermal heating, the high electrical conductivity and low infrared emissivity of MXenes serve as the basis for energy-efficient wearable heaters, deicing devices, crude oil separation, and soft actuators for active thermal regulation. Finally, we discuss unresolved challenges and propose future research directions, offering a framework to guide the advancement of next-generation MXene-based thermal management platforms that integrate fundamental insights with practical, energy-efficient implementation.

## 2 Fundamental Properties of MXenes

MXenes exhibit low emissivity with high electrical conductivity in the in-plane direction, while demonstrating low thermal conductivity in the out-of-plane direction, thereby not adhering to the Wiedemann–Franz law applicable to most metals.^32,33^ These unusual characteristics arise from the structural anisotropy of 2D MXenes, which facilitate efficient electron and heat transport along the in-plane direction through robust intraflake pathways,^34^ while the interlayer spacing in the out-of-plane direction substantially suppresses heat transfer, as illustrated in Scheme 1. Furthermore, strong interband transitions in the ultraviolet–visible-near-infrared (UV–vis-NIR) spectrum render MXene a highly efficient solar light absorber, supporting a range of photothermal applications, such as water desalination and biomedical imaging.^6^ Moreover, the adjustability of chemical configuration in MXenes permits precise tailoring of optical and thermal properties, supporting property-oriented atomistic engineering.^9^

**Scheme 1:**
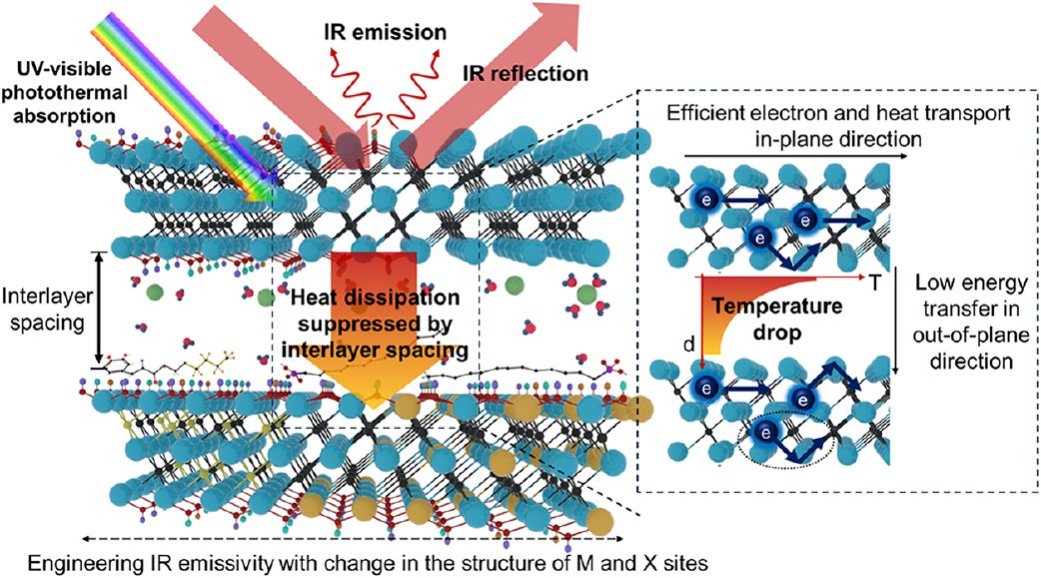
Schematic Illustrating the Interaction of IR Light with MXenes, and Heat Transfer Mechanisms Coupled to Structure Parameters in MXenes^a^

a The atomic composition of MXene, including the transition metal, carbon, nitrogen, and surface terminations, plays a crucial role in determining IR emissivity and reflectivity, which are linked to conductivity, as well as UV–visible photothermal absorption. The interlayer spacing present in MXenes, which is tunable through surface terminations modification, intercalant insertion, or introducing structural anisotropy. As a result, MXenes typically exhibit low thermal conductivity in the out-of-plane direction, while retaining high in-plane thermal and electrical conductivity due to an effective free electron response through intraflake pathways.

Specifically, MXenes offer diverse layered-structure configurations with varying single-transition-metal-to-carbon/nitrogen ratios, including M_2_XT_*x*_, M_3_X_2_T_*x*_, M_4_X_3_T_*x*_, and M_5_X_4_T_*x*_. In addition, multielement transition metal compositions—including solid-solution MXenes, *i*-MXenes featuring in-plane ordered transition metal, and *o*-MXenes with out-of-plane ordered M layers—further expand possibilities for atomistic design.^35,36^ Tailoring these configurations enables precise control of thermal properties, such as emissivity modulation over a wide range of electrical conductivities, as well as optical properties, including photothermal absorption across different spectral ranges.

One of the distinguishing features of MXenes is their surface terminations, which are determined by synthesis methods and can substantially influence their material properties. Wet chemical etching of the MAX phase in fluoride or chloride-based solutions produces mixed surface terminations on the MXene surface, including −F, −OH, −Cl, and −O. In contrast, molten salt and dry gas-phase etching synthesis, or direct vapor-phase growth of MXenes, can yield more uniform surface terminations, such as halogens or chalcogens. Subsequent chemical treatments can graft organic terminations on MXenes. Engineering surface terminations alters the electronic band structure and modulates optical characteristics, such as the position of the absorption peaks within the UV–visible spectrum.^37^ Moreover, termination design can be used to tailor interlayer spacing, thereby controlling out-of-plane thermal conductivity. Notably, MXenes with organic terminations, which possess longer molecular chains compared to single-element terminations, can suppress thermal dissipation across enlarged interlayer spacing, leading to reduced out-of-plane thermal conductivity as illustrated in Scheme 1.

An alternative approach for tuning interlayer spacing is the intercalation of various mineral or large organic molecule cations. For instance, introducing organic molecules such as trimethylamine (TMA) or cetyltrimethylammonium bromide (CTAB) expands the interlayer spacing of Ti_3_C_2_T_*x*_ MXene from 0.26 nm (following LiCl intercalation) to 1.23 nm (with CTAB intercalation). In addition, inorganic cations such as Na, K, Ca, Cs, or Mg regulate interlayer distance according to cation size and also alter the microenvironment through water confinement effects. The extent of water confinement in MXene films depends on the hydration degree of the intercalated cation, with more hydrophilic cations generally producing greater interlayer spacing due to strong electrostatic interactions with water molecules.^38^ This section discusses how these structural parameters in the chemical configuration influence the optical and thermal behaviors of MXenes.

### 2.1 Optical Properties: Spectral Response and Tunability

MXenes possess distinctive optical spectral properties, including broadband absorption, selective light absorption, and inherently low infrared emissivity. Additionally, their optical and thermal characteristics can be precisely tailored by controlling composition and surface terminations, enabling spectral modulation from the ultraviolet to the mid-infrared.^9^ Figure 2(a) demonstrates that each MXene colloidal solution and its respective MXene film exhibits a diverse color attributed to different interband transitions in the UV–visible region.^39^ Furthermore, even for MXenes comprising the same elements, the hue of the films can be precisely adjusted by varying the M and X stoichiometry or element ratios, as depicted in Figure 2b.^40^ The correlation between the atomic compositions at the M and X sites in MXenes and their absorption characteristics is presented in the complete UV–vis-NIR transmission spectra of different MXene films (Figure 2c).^39^ Although various MXene films display distinctive spectral signatures, with interband transition peaks in the UV–vis range, most show either flat or monotonically decreasing transmittance at wavelengths above 1000 nm. This reduction in transmittance for MXenes at lower frequencies (longer wavelengths) is attributable to the Drude response of free electrons. In particular, Ti_3_C_2_T_*x*_ MXene exhibits an epsilon-near-zero (ENZ) point, where the permittivity approaches zero within the NIR region, exhibiting metallic behavior with negative permittivity above the ENZ point and demonstrating strong absorption below 1000 nm (Figure 2d).^41^ Similar optical phenomena are also observed in titanium carbonitride and Ti_2_CT_*x*_ MXenes, suggesting that those optical properties can be correlated to metal and carbon/nitrogen bonding natures.

**Figure 2:**
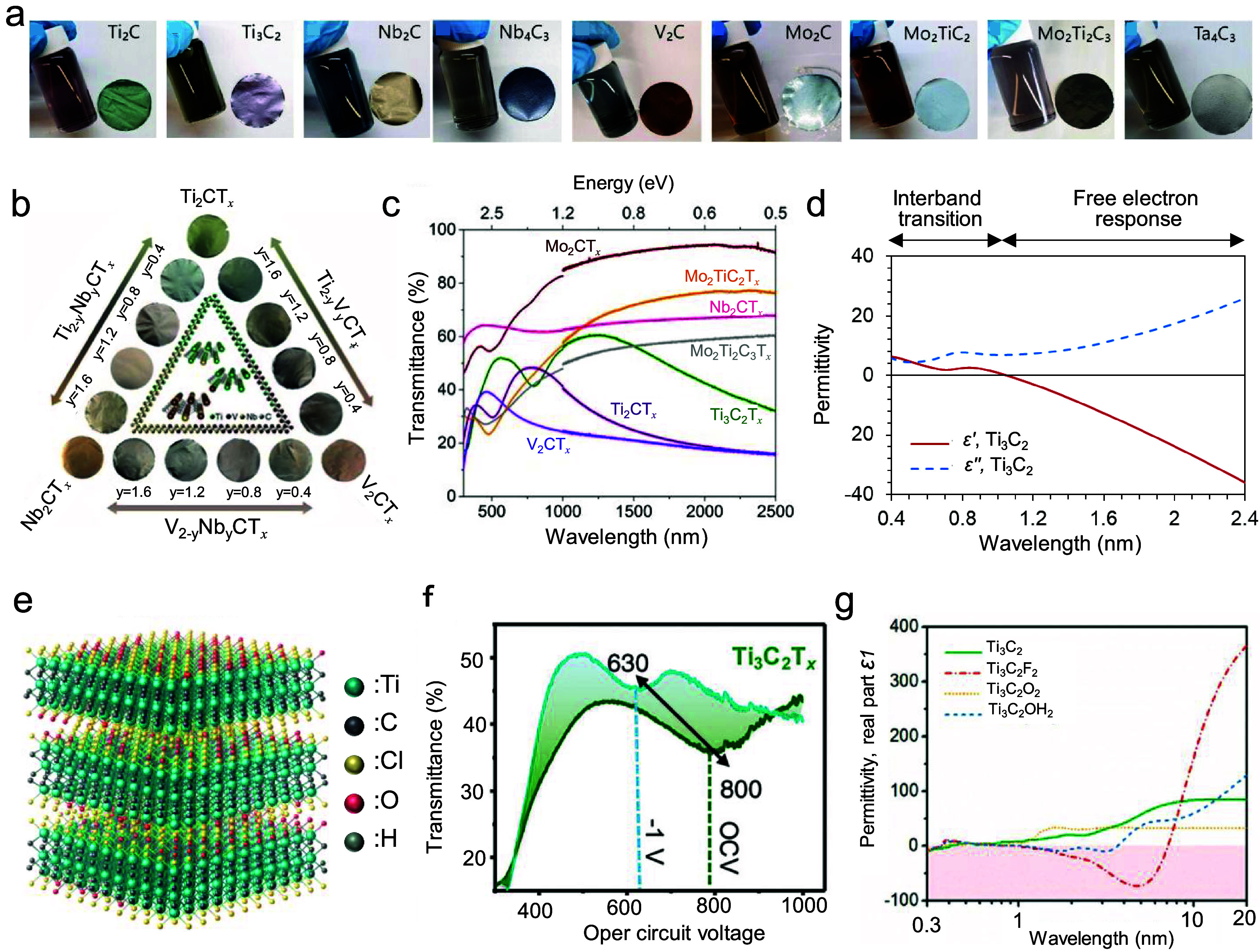
Optical properties of MXenes. (a) Digital photographs of colloidal solutions of different MXenes and their corresponding freestanding films. Reproduced with permission.^39^ Copyright 2020, John Wiley and Sons. (b) Freestanding film coloration of M′_2–*y*_M″_*y*_CT_*x*_ MXenes with precisely controlled stoichiometry. Reproduced with permission.^40^ Copyright 2020, American Chemical Society. (c) UV–vis-NIR transmittance spectra of colloidal solutions with various MXenes. Reproduced with permission.^39^ Copyright 2020, John Wiley and Sons. (d) Measured real and imaginary permittivity curves of Ti_3_C_2_T_*x*_ MXene in the UV–vis-NIR range. (e) Schematic representation of Ti_3_C_2_T_*x*_ MXene showing mixed surface terminations, including −OH, –O, and –F groups. Reproduced with permission.^42^ Copyright 2022, Springer Nature. (f) In situ modulation of transmittance using electrochemical approaches by applying an electrical potential relative to open circuit voltage. Reproduced with permission.^43^ Copyright 2020, Royal Society of Chemistry. (g) Calculated permittivity curves for Ti_3_C_2_T_*x*_ MXene with different surface terminations. Reproduced with permission.^6^ Copyright 2021, John Wiley and Sons.

Surface terminations also impact the optical properties of MXenes, though their effect is generally less pronounced than that of compositions at the M and X sites. As shown in Figure 2e, MXenes such as Ti_3_C_2_T_*x*_ synthesized via wet chemical etching methods, typically possess mixed terminations (T = O, OH, F, and Cl), and the type of termination can be modified through variations in the etching protocol or through postprocessing methods like electrochemical redox treatments.^42^ Figure 2f demonstrates how surface terminations influence optical characteristics as a function of the applied electrical potential in electrochemical redox processes. At open circuit voltage (OCV), most surface terminations on Ti_3_C_2_T_*x*_ are expected to be oxygen due to the oxidation process, resulting in an absorption peak centering at low photon energies (1.55 eV). When a −1 V potential is applied, the reduction process in Ti_3_C_2_T_*x*_ results in surfaces rich in hydroxyl terminations, leading to a blueshift of the absorption peak center.^43^

Figure 2g presents the calculated permittivity curves for Ti_3_C_2_T_*x*_ with various surface terminations, highlighting their role in defining the optical properties of MXenes.^6^ Experimental studies have revealed the plasmonic characteristics of Ti_3_C_2_T_*x*_ across the mid-infrared (MIR) spectral region, characterized by negative permittivity. However, different surface terminations can alter key optical features, including the ENZ point, plasma frequency, and tangent loss. In particular, hydroxyl-terminated surfaces are generally associated with higher plasma frequencies and greater electron densities compared to oxygen-terminated ones, as evidenced by an in situ redox experiment shown in Figure 2f.^43^

The optical properties of MXene in the MIR range are closely associated with thermal radiation. According to Kirchhoff’s law of energy conservation, the emissivity of MXenes is defined as

1 ε=α=1−ρ−τ

Under thermal equilibrium, where ε, α, ρ, and τ represent emissivity, absorptivity, reflectivity, and transmissivity, respectively, the behavior of metallic films can be effectively described. The sum of these parameters equals one, and each is a dimensionless quantity ranging from 0 to 1. For thick metallic films, emissivity can be estimated from the reflectance spectrum since transmittance is nearly zero, as shown in Figure 3a.^10^ The emissivity of various MXenes was determined from their reflectance spectra in the MIR range (Figure 3b). In metallic films, the free electron response becomes dominant at low frequencies, resulting in high reflectance. Accordingly, all the metallic MXene films in Figure 3b exhibit a monotonically decreasing trend in emissivity, accompanied by strong reflectance. Lower-conductivity MXene films exhibit greater skin depth, which reduces reflection and increases emissivity, whereas higher conductivity produces lower emissivity, as shown in Figure 3c.^10^ Although optical and electrical conductivity are distinct, they are proportional in metallic films according to the Drude model, expressed as σ_AC_(ω) = σ_DC_/(1 – iωτ_0_), where σ_AC_ and σ_DC_ are the optical and electrical conductivities, respectively, and τ_0_ is the electron collision time. This relationship accounts for the clear correlation between electrical conductivity and emissivity observed in Figure 3c. Because the optical conductivity of MXenes is strongly influenced by the chemical composition at both the M and X sites, their emissivity can be tuned from 0.06 (for Ti_3_C_2_T_*x*_) to 0.59 (for Nb_2_CT_*x*_), as shown in Figure 3d.^10^ This tunability enables a broad spectrum of thermal applications, from thermal insulation with very low emissivity to highly efficient thermal emitters. Moreover, the emissivity range for individual MXene films can be further extended by modifying interlayer spacing or surface terminations.^9^ Remarkably, MXenes can achieve this wide emissivity range even at very small thickness (200 nm), outperforming other materials shown in Figure 3d.

**Figure 3:**
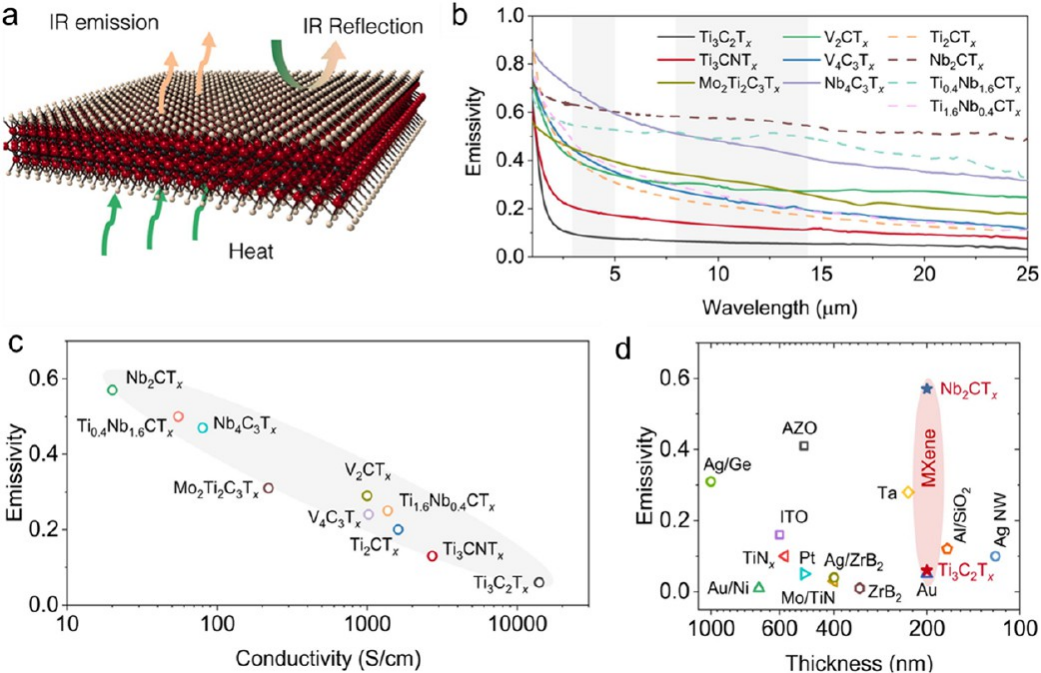
Emissivity and heat loss characteristics of MXenes. (a) Illustration depicting the interaction of IR radiation with heated MXenes. (b) Experimental emissivity values of different MXenes, each with a thickness of 200 nm. (c) Correlation between emissivity and electrical conductivity for MXenes. (d) Comparative plot presenting the emissivity of various materials as a function of thickness. Reproduced with permission.^10^ Copyright 2023, Elsevier.

Polished metals are widely recognized for their high reflectivity across a broad spectral range, attributed to their electron oscillation with Drude response (Figure 4a). On the contrary, carbon-based absorbers, strongly absorb across the UV–visible–IR spectrum with high absorption/emissivity (Figure 4b). For instance, a carbon nanotube (CNT) black absorber, for instance, demonstrates a high solar absorptance (α) of up to 95% and high IR emissivity (ε) of 93%.^6^ In contrast, MXenes, particularly Ti_3_C_2_T_*x*_ MXene, displays a high α of 90% in UV–vis and near IR region,^6^ comparable to that of CNT absorbers, while showing extremely low IR emissivity, similar to that of metals (Figure 4c). Its absorption sharply decreases outside the solar spectrum due to Drude response being more dominant than Lorentz response, thereby leading to increase of reflection in mid-IR range (Figure 1c), indicating excellent spectral selectivity (α/ε).

**Figure 4:**
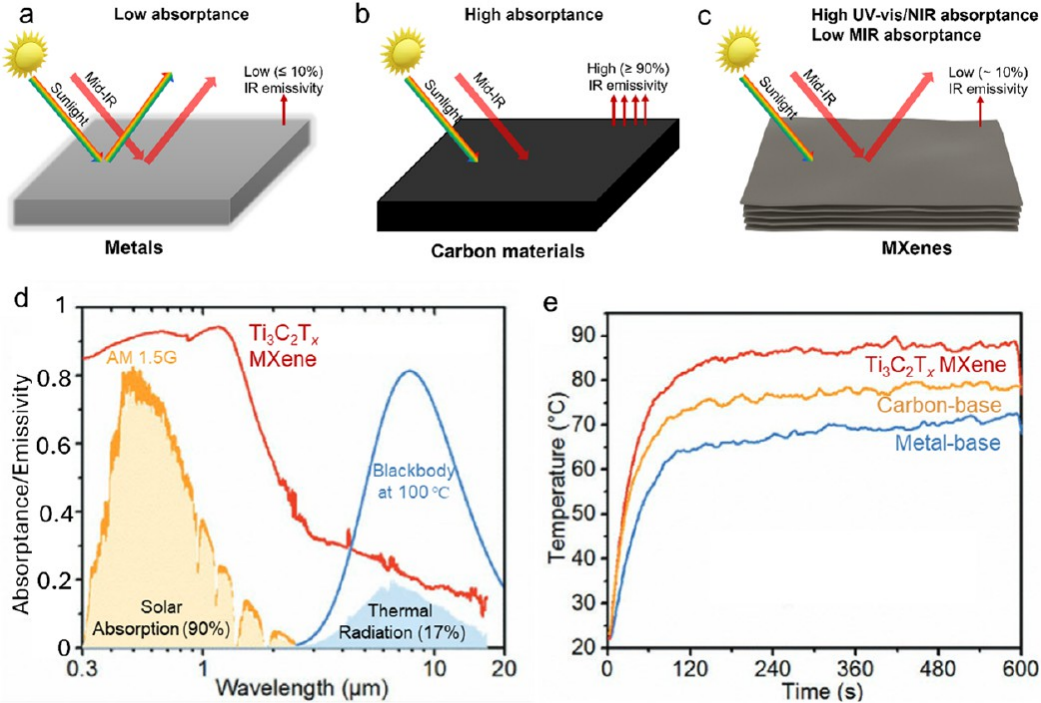
Light–matter interaction of metals, carbon-based materials, and MXenes. (a) The low solar and mid-IR absorptance, and low IR emissivity of metals. (b) The high solar and mid-IR absorptance, and high IR emissivity of carbon-based blackbodies. (c) The high solar absorptance, low mid-IR absorptance, and low IR emissivity of MXenes. (d) Absorptance/emissivity spectra of Ti_3_C_2_T_*x*_ MXene film in a broad range from UV to mid-IR. The yellow and blue shaded areas indicate absorbed energy from the air mass 1.5 global (AM 1.5G) solar spectrum and thermal radiation at 100 °C in comparison to a blackbody, respectively. (e) Temperature evolution over time resulting from the photothermal effect in Ti_3_C_2_T_*x*_ film (red curve), carbon-based black paint film (yellow curve), and metal-based metamaterials (blue curve) when exposed to 1 sun illumination. Reproduced with permission from ref 6. Copyright 2021 John Wiley and Sons.

MXenes possess distinctive optical characteristics, featuring high absorption in the UV–visible and NIR regions but displaying low emissivity in the MIR range (Figure 4d).^6^ This combination allows for efficient photothermal conversion with reduced radiative heat loss. In contrast, many conventional low-emissivity materials, such as noble metals, show low solar absorptance, while blackbody materials like carbon-based black paint absorb solar energy efficiently but show very high IR emissivity. Ti_3_C_2_T_*x*_ MXene combines pronounced interband transitions with Drude-like free electron responses, supporting efficient solar absorption and photothermal heating under sunlight, while low IR emissivity leads to minimizing thermal radiation losses.

Figure 4e shows that Ti_3_C_2_T_*x*_ MXene films exhibit superior photothermal behavior compared with both carbon-based black paint and metal-based metamaterial under a light intensity of 1000 W m^–2^ (1 Sun).^6^ Although carbon-based black paint is typically considered an ideal blackbody material for photothermal effects due to its near-unity absorption (∼100%) across the solar spectrum, its high emissivity throughout the mid-IR range leads to significant radiative heat loss. In contrast, Ti_3_C_2_T_*x*_ MXene absorbs slightly less solar energy than carbon-based black paint but possesses exceptionally low IR emissivity, allowing it to retain the generated heat more effectively and achieve a higher temperature rise under identical sunlight conditions. These attributes make MXenes promising for a range of thermal applications, including photothermal conversion,^44^ multispectral camouflage,^45^ and solar energy harvesting.

### 2.2 Electrical and Thermal Transport Properties

MXenes exhibit a combination of high in-plane electrical conductivity and notable transport anisotropy due to their metallic character and two-dimensional layered configuration, making them ideally suited for advanced thermal management applications. Owing to their flake-like structure, MXene films are commonly assembled with two-dimensional flakes aligned parallel to the substrate, which favors in-plane characterization of their electronic properties for performance assessment. Multiple variables, such as atomic composition, crystalline structure, restacking arrangements, and synthesis protocols—including etching, intercalation, and delamination—affect the electrical conductivity of MXene thin films. The electrical conductivity values of MXene films span a broad range, from 5 to 8570 S cm^–1^ (Figure 5a),^46^ indicating strong dependence on the atomic composition and crystalline structure. The highest electrical conductivity reported is 24,000 S cm^–1^ for Ti_3_C_2_T_*x*_ MXene,^47^ while a recent investigation claimed an even higher conductivity for Ti_3_C_1.75_N_0.25_T_*x*_ MXene by optimizing both the elemental composition, synthesis conditions, and thermal treatment parameters.^48^

**Figure 5:**
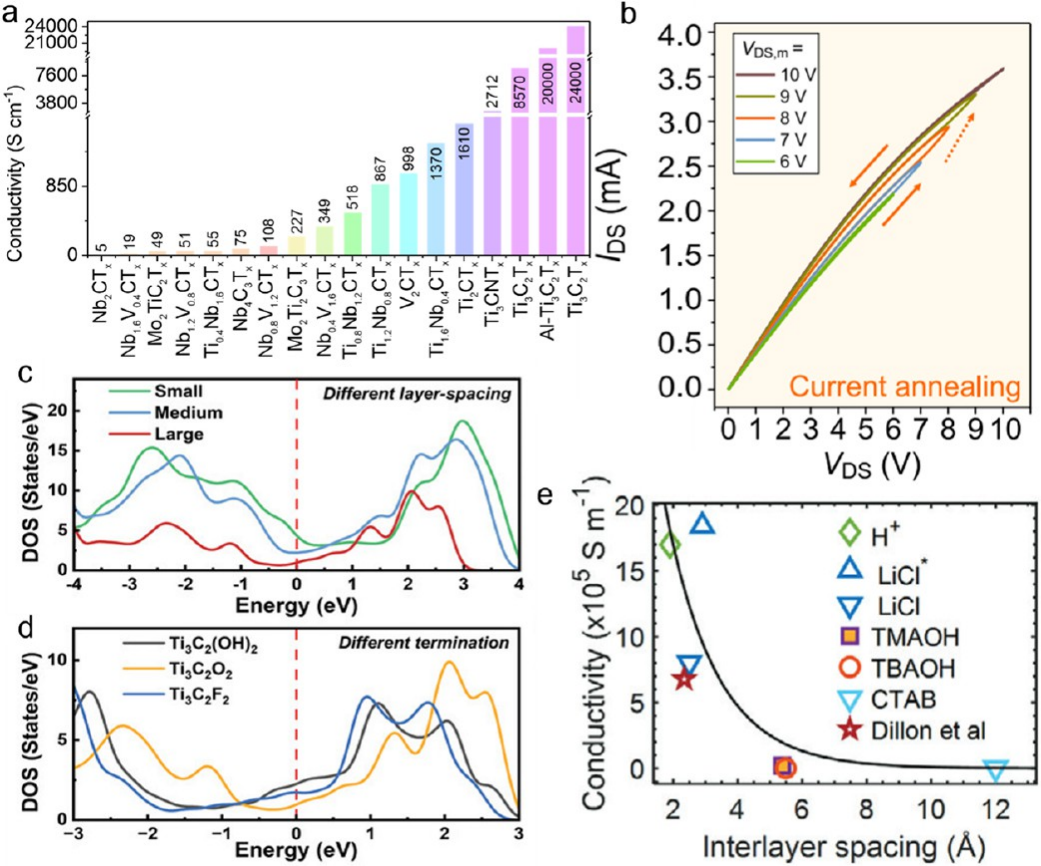
(a) In-plane electrical conductivity values for several types of MXenes. Reproduced with permission.^46^ Copyright 2020, American Chemical Society. (b) *I–V* curves measured under varying applied voltages. The progressive variation highlights the influence of Joule heating. Reproduced with permission.^50^ Copyright 2021, Elsevier. (c, d) Calculated density of states (c) for Ti_3_C_2_O_2_ MXene at different interlayer spacings, and (d) for Ti_3_C_2_T_*x*_ MXene with distinct surface terminations (−OH, –O, and –F). Reproduced with permission.^52^ Copyright 2023, John Wiley and Sons. (e) Dependence of electrical conductivity in Ti_3_C_2_T_*x*_ MXene on interlayer spacing, modulated by the introduction of different intercalants. Reproduced with permission.^53^ Copyright 2024, John Wiley and Sons.

Thermal post-treatment is a crucial parameter, particularly when intercalants are present, as these species can markedly influence both carrier transport and interlayer spacings. For example, TBA^+^-intercalated Mo_2_CT_*x*_ MXene initially exhibited a low conductivity of 0.8 S cm^–1^, which dramatically increased to 1252 S cm^–1^ after annealing at 800 K.^49^ Upon thermal treatment, the intercalated molecules are decomposed and OH surface terminations are removed. Such changes not only reduce the interlayer spacing but also result in switching from *p*-type to *n*-type conduction characteristics in Mo_2_CT_*x*_ MXene. Hall-effect measurements confirmed that the substantial electrical conductivity in MXene is mainly attributed to a high electron density rather than mobility, which remains quite low, typically just a few cm^2^ V^–1^ s^–1^. In restacked MXene films, carrier transport is frequently hampered by interflake resistance. Notably, localized Joule heating occurring during device operation can enhance carrier transport by as much as 20% (Figure 5b), a phenomenon likely explained by the removal of surface-adsorbed molecules and photolithography residues.^50^

Theoretical predictions indicate that most MXenes display metallic electronic band structures, with their density of states persisting at the Fermi level. Nevertheless, some “thinnest MXenes” with M_2_C stoichiometry and oxygen functionalization are anticipated to possess bandgaps. Despite these theoretical expectations, experimental evidence, such as observation of gate-tunable transport behavior within a transistor architecture, remains absent.^51^ In comparison, “thicker MXenes” like M_3_C_2_ and M_4_C_3_ exhibit a greater number of electronic bands, making it less feasible for them to display a bandgap. Computational models further reveal that both surface chemistry and interlayer separation in Ti_3_C_2_T_*x*_ MXene considerably impact its electronic density of states (Figure 5c,d).^52^ However, there is often a lack of consistency between computational predictions and experimental observations, especially regarding properties dependent on layer thickness or quantum confinement phenomena. Such inconsistencies can be attributed to factors including heterogeneous surface functionalization, existing defects, and intercalant incorporation in real materials. Intercalation generally leads to greater interlayer separation, which in turn reduces electrical conductivity (Figure 5e).^53^

Uniform surface termination has been reliably achieved via the Lewis acid molten salt etching method.^54^ This technique has also been extended to the fabrication of MXenes exhibiting uniform halogen and chalcogen terminations and to the synthesis of MAX phases with A-element substitution.^55^ Accurate manipulation of surface terminations has facilitated the observation of superconductivity in Nb_2_C MXene with Cl_2_, S_2_, Se, or NH surface groups.^37^ Furthermore, Ti_3_C_2_Cl_2_ MXene exhibits an electrical conductivity of 8000 S cm^–1^ and outstanding oxidation stability, retaining 90% of its conductivity after 2 weeks of exposure to a high-humidity (95%) environment.^56^

The 2D morphology of MXenes promotes preferential electronic and thermal transport along the in-plane direction, while out-of-plane transport is rather limited. Such anisotropic transport is attributed to the heterogeneity of van der Waals stacking and the random orientation between adjunct flakes. Additionally, the incorporation of intercalants such as ions and molecules can modulate these anisotropic transport characteristics.

Due to this anisotropy, Ti_3_C_2_T_*x*_ MXene shows extremely high thermal resistance (7.1 MK W^–1^), which is higher than that of SiO_2_ (6.3 MK W^–1^) known as thermal insulator, as observed in a scanning thermal microscopy (SThM) measurement (Figure 6a). Compared to the in-plane direction, out-of-plane thermal energy transport by electrons and phonons is largely suppressed due to scattering in the interlayer spacing in MXenes, as illustrated in Figure 1. This restriction of electron and phonon movement in the out-of-plane direction allows ultralow thermal conductivity (0.38–0.63 W m^–1^ K^–1^) in the Ti_3_C_2_T_*x*_ monolayer. We note that this thermal conductivity value can be tuned by controlling not only M and X elements but also interlayer spacing with various methods, including intercalation with large molecules or change of surface termination, which can directly engineer heat transfer in the out-of-plane direction.

**Figure 6:**
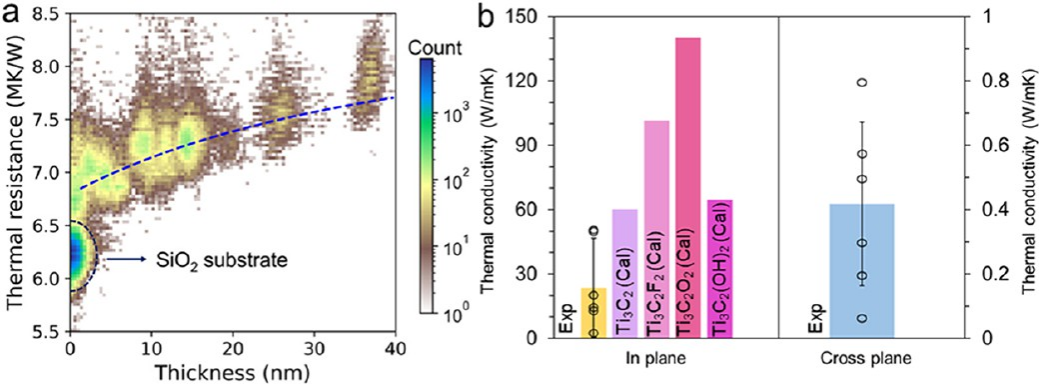
(a) Thermal resistance of Ti_3_C_2_T_*x*_ MXene as measured by scanning thermal microscopy (SThM) is shown. Ti_3_C_2_T_*x*_ demonstrates lower thermal conductivity than SiO_2_. Reproduced with permission.^33^ Copyright 2024, American Chemical Society. (b) In-plane and out-of-plane thermal conductivities of Ti_3_C_2_T_*x*_ MXene from experimental data and theoretical calculations are reported, revealing substantial discrepancies and significant variability depending on the methodology.^32,33,57–64^

In addition to the SThM technique, thermal conductivity measurements of Ti_3_C_2_T_*x*_ have been reported with direct methods, such as laser flash and pump–probe techniques, and indirect methods (e.g., Raman signal based estimation). Figure 6b summarizes reported thermal conductivity in the in-plane and out-of-plane directions. Even if the value of thermal conductivity largely depends on measurement technique, it is observed that thermal conductivity in the in-plane direction is approximately 2 orders of magnitude higher than that in the out-of-plane direction. When we take the value of electrical conductivity Ti_3_C_2_T_*x*_ into account, such a high in-plane thermal conductivity obviously deviates from theoretical prediction by Wiedemann–Franz law. This illustrates that thermal energy transport in in-plane direction is governed by phonon scattering while electrical conductivity is attributed to electron movement.^33^

According to theoretical predictions, MXene could reveal a wide range of in-plane thermal conductivity values with different surface terminations. For instance, at room temperature, the calculated in-plane thermal conductivity of Ti_3_C_2_F_2_ is 108.2 W m^–1^ K^–1^, while that of Ti_3_C_2_(OH)_2_ is much lower at 64.6 W m^–1^ K^–1^.^57,58^ This difference is associated with a change of electronic properties with surface terminations, such as electron density of states, as shown in Figure 2f,2g. In the experimental results, because MXene obtained with the wet chemical etching method generally has mixed surface termination, electron and phonon scattering due to nonuniform medium can contribute to measured thermal conductivity much lower than the theoretical prediction in Figure 6b.

In Figure 6b, a large discrepancy in thermal conductivity is observed between measurement results. A micro-Raman-based approach estimated the thermal conductivity of multilayer Ti_3_C_2_T_*x*_ MXene to be 15.38 W m ^–1^ K^–1^.^59^ The laser flash technique determined the in-plane and out-of-plane thermal conductivities of Ti_3_C_2_T_*x*_ MXene to be 55.5 and 1.44 W m^–1^ K^–1^, respectively.^60^ It is noteworthy that the laser flash method may result in an overestimation of thermal diffusivity and generally requires millimeter-thick samples for obtaining accurate measurements. Ultrafast transient absorption spectroscopy was employed to investigate carrier dynamics of MXene thin films.^61,62^ By fitting the pump–probe results using a one-dimensional thermal diffusion model, the out-of-plane thermal diffusivity of Ti_2_CT_*x*_ and Ti_3_C_2_T_*x*_ was determined to be 0.06 and 0.02 mm^2^ s^–1^, respectively. The value for Ti_3_C_2_T_*x*_ is 21 times lower than values obtained through the laser flash technique (0.422 mm^2^ s^–1^). The experimental and theoretical thermal conductivity values of MXenes, compiled from the literature, are presented in Figure 6b, illustrating the wide variation in reported thermal conductivities across different studies.^32,33,57–60,63,64^ Such discrepancies are attributed to variations in sample density and heat capacity arising from different synthesis protocols, but also different assumptions and models with the measurement technique. Establishing standardized synthesis and measurement approaches is critical to facilitate reliable comparisons across various studies.

### 2.3 Comparison of MXenes with Conventional Materials

The thermal properties of Ti_3_C_2_T_*x*_ MXene and other representative materials are compared in Figure 7. Applications such as zero-energy buildings and thermal camouflage require materials that simultaneously exhibit low thermal conductivity (to hinder heat conduction) and low emissivity (to minimize thermal radiation loss). However, conventional materials rarely satisfy both requirements.

**Figure 7:**
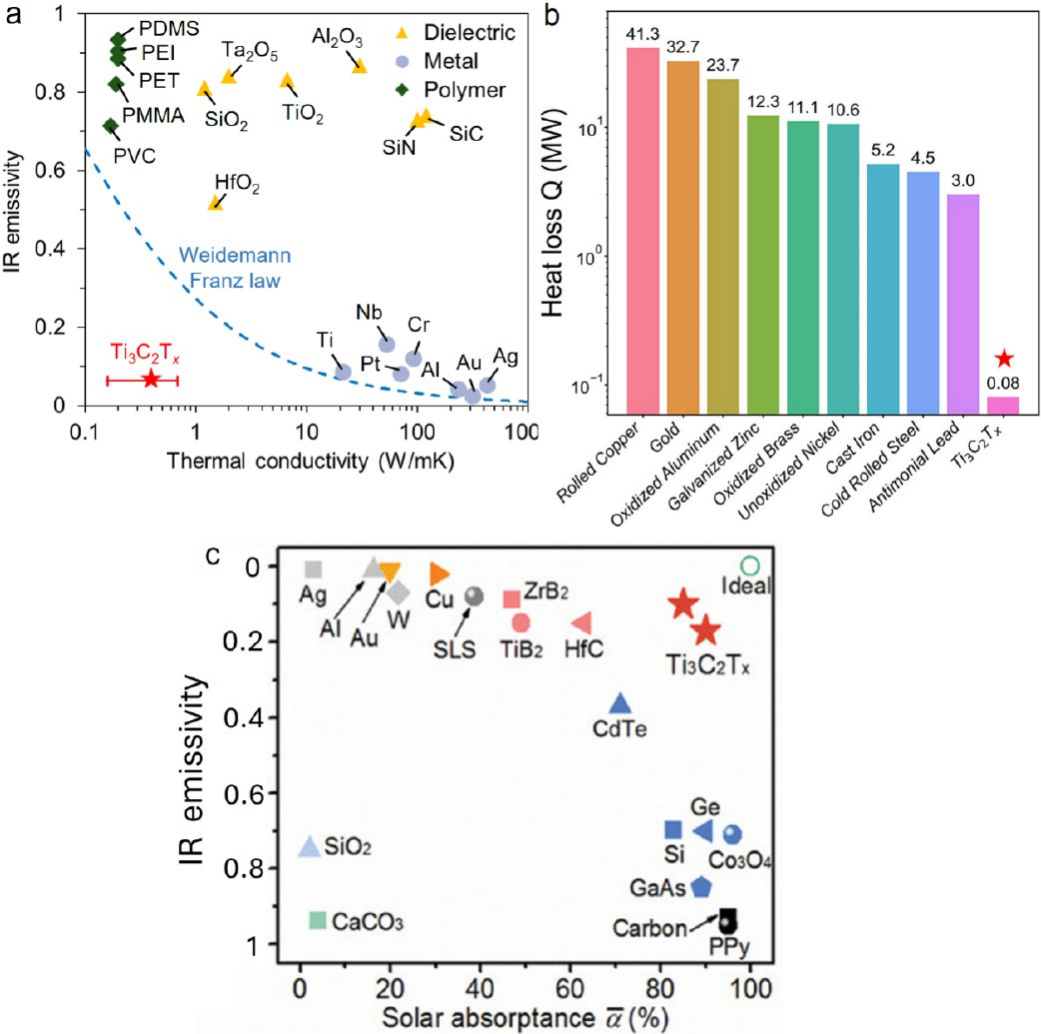
Comparison of thermal properties for several reported material systems. (a) IR emissivity in long-wave infrared range versus thermal conductivity. For emissivity of dielectric and polymer, we calculated the values with refractive index taken from the literature, assuming film thickness as 10 μm, and we adopted the emissivity value of metal and Ti_3_C_2_T_*x*_ MXene from the literature. The blue dotted line denotes the expected correlation as per the Wiedemann–Franz law at temperature of 300 K. To evaluate emissivity from electric conductivity in Wiedemann–Franz law, we used Drude model with a scattering time of 15 fs, and frequency of 30 THz.^10,33,65–71^ (b) Relative estimation of heat loss under conditions relevant to thermal shielding applications. Reproduced with permission.^33^ Copyright 2024, American Chemical Society. (c) IR emissivity plotted against solar absorptance, illustrating intrinsic trade-offs in photothermal material design. Reproduced with permission from ref 6. Copyright 2021 John Wiley and Sons.

Figure 7a displays the comparison of thermal conductivity and emissivity among different materials. In metals, both electrical and thermal conduction predominantly occur through free electron movement, resulting in a direct relationship between thermal and electrical conductivity as described by the Wiedemann–Franz law. As a result, metals typically display low infrared emissivity but high thermal conductivity, as shown in Figure 7a, which is calculated from optical properties of materials.^10,33,65–71^ In contrast, dielectric or nonmetallic materials such as SiO_2_ or Al_2_O_3_ possess low thermal conductivity due to the absence of free electron carriers, but exhibit high infrared emissivity caused by strong phonon dispersion or molecular vibrations in the MIR range.

MXenes offer a distinct advantage—their anisotropic structure enables the simultaneous suppression of both heat conduction and thermal radiation. In MXenes, in-plane optical conductivity governs emissivity, while out-of-plane thermal transport remains extremely low owing to interflake barriers and the increased interlayer spacing, both of which impede out-of-plane heat transfer. This anisotropic behavior positions Ti_3_C_2_T_*x*_ MXene in the lower-left region of the plot in Figure 7a, approaching the ideal characteristics for thermal insulation applications.^72^

To assess insulation capability, heat loss was calculated taking into account both thermal conductivity and emissivity (Figure 7b).^33^ The calculation assumes a 1 mm thick film, 1 m^2^ surface area, a heating temperature of 395 K, and an ambient temperature of 295 K. Under these conditions, Ti_3_C_2_T_*x*_ MXene achieves a total heat loss of 0.08 MW, which is at least 37.5 times smaller than that of common metals.^33^ Improved performance can be achieved by tuning the interlayer spacing, which controls thermal conductivity through methods such as intercalation or engineering of surface terminations.

A comparison of the photothermal performance of reported materials is presented in Figure 7c.^6^ Metals predominantly reflect incident solar light owing to free electron oscillations, which restricts their photothermal conversion efficiency. Dielectric materials and semiconductors (e.g., carbon black, HfC) absorb solar energy through bandgap or interband transitions; however, their high emissivity leads to significant thermal losses. By contrast, Ti_3_C_2_T_*x*_ exhibits strong interband absorption in the UV–vis–NIR range alongside a broad free-electron response in the MIR, resulting in effective photothermal conversion and minimal thermal radiation loss. This synergistic behavior highlights MXenes as highly promising candidates for solar–thermal applications where retention of generated heat is essential.

## 3 MXenes for Thermal Insulation and Thermal Camouflage

### 3.1 Mechanism of Thermal Camouflage

A notable potential application of MXenes lies in their capacity to modulate heat transfer simultaneously via conduction and radiation, thereby positioning them as attractive materials for both thermal insulation and thermal camouflage. To emphasize the distinctive advantages of MXenes, the heat transfer mechanisms in three representative material classes—metals, porous insulators, and MXenes—are contrasted in Figure 8a. In metals, high optical conductivity arising from the Drude response of free electrons results in exceptionally low emissivity, typically below 0.05 for gold. Nonetheless, the same Drude (free electron) response in metals is also responsible for high thermal conductivity, leading to pronounced heat loss. Porous insulators, such as aerogels, are widely adopted as thermal insulators due to their extremely low thermal conductivity (<0.1 W m^–1^ K^–1^), which is mainly attributed to the presence of air-filled pores impeding heat transfer. However, this reduction in thermal conductivity is offset by low electrical and optical conductivity and correspondingly high emissivity. Moreover, the substantial fraction of air within the porous matrix strongly compromises mechanical integrity, rendering these structures highly brittle and susceptible to fracture.

**Figure 8:**
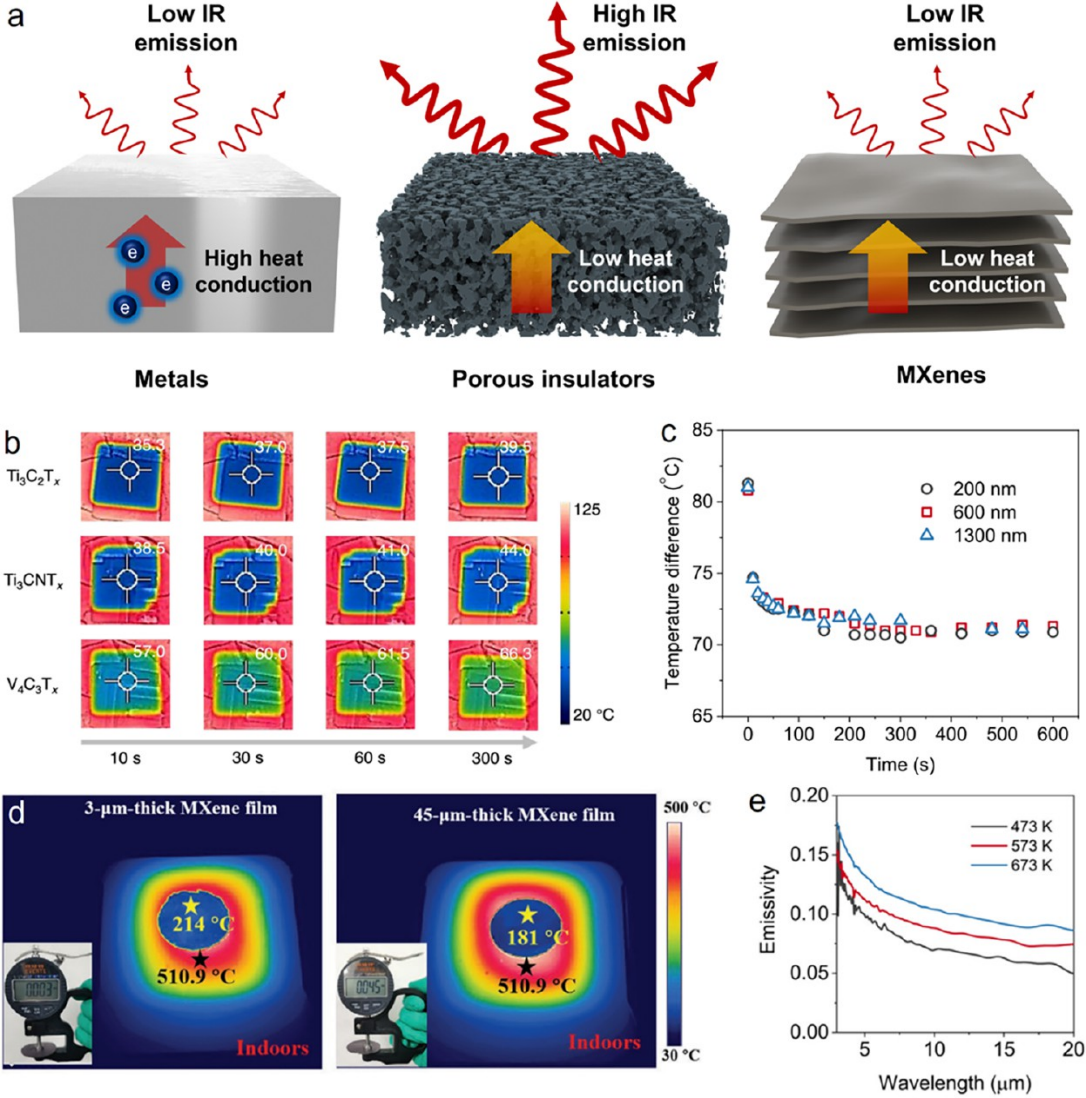
Thermal camouflage characteristics of MXenes. (a) Schematic depiction of the thermal camouflage mechanisms for three scenarios: metals, porous insulators, and MXenes. (b) IR surface temperature of different MXene films on a hot plate as a function of heating duration. The hot plate was maintained at 110 °C, and the film thickness was 200 nm. (c) Temperature curves of various MXene films with different thicknesses over time, measured on a hot plate at 110 °C. Reproduced with permission.^10^ Copyright 2023, Elsevier. (d) IR surface temperature of free-standing Ti_3_C_2_T_*x*_ films with different thicknesses at a heating temperature of 510 °C. Reproduced with permission.^29^ Copyright 2021, John Wiley and Sons (e) Temperature-dependent IR emissivity profiles of Ti_3_C_2_T_*x*_ films. Reproduced with permission.^10^ Copyright 2023, Elsevier.

In contrast, MXenes exhibit intrinsic anisotropy in electron transport, characterized by high in-plane conductivity and low out-of-plane conductivity, which enables simultaneous suppression of both thermal conduction and radiation. In the out-of-plane direction, heat transfer is primarily regulated by phonon–phonon interactions and electron scattering within the conducting layers, while the interlayer spacing inherent to MXenes increases phonon scattering by introducing phonon dispersion mismatch, resulting in limited out-of-plane thermal conductivity. Meanwhile, electrons constrained within the geometry of MXene flakes predominantly migrate along the in-plane axis, which assists in effectively screening IR light through in plane oscillations. This electron-driven screening reduces IR absorption and yields low emissivity, which is determined by the mobility and density of electrons and can be precisely tuned via chemical modification and surface termination.

The relationship between MXene composition and thermal radiation characteristics is illustrated in Figure 8b.^10^ In ultrathin films (∼200 nm), radiative heat transfer significantly outweighs conductive mechanisms in determining the IR temperature. MXenes provides a broad range of emissivity values, spanning from 0.06 to 0.59 (see Figure 3d), which facilitates customization for thermal camouflage by aligning IR profiles with diverse backgrounds. As presented in Figure 8c, increasing the MXene film thickness from 200 nm to micron scales does not further diminish thermal radiation, indicating that 200 nm is sufficient to block IR transmission and achieve low emissivity.^10^ Importantly, despite their minimal thickness, MXene films demonstrate significantly slower thermal equilibration times compared to noble metals—remaining in the tens of seconds range—mainly due to their restrained thermal conductivity as depicted in Figure 8c.

Interestingly, the contribution of thermal conduction becomes notable in MXene films with thicknesses reaching several tens of micrometers, as indicated in Figure 8d.^29^ At 500 °C, a 45 μm-thick free-standing Ti_3_C_2_T_*x*_ film achieves an IR surface temperature of 181 °C, approximately 30 °C lower than that of its thinner counterparts. For comparison, quartz—recognized for its insulating properties with a thermal conductivity of 1.4 W m^–1^ K^–1^—requires millimeter-scale thicknesses to establish a comparable temperature gradient. The ability of MXenes to display significant thermal conduction effects at micron-scale thicknesses underscores their promise as ultrathin materials for thermal camouflage.^29^

In the development of high-temperature thermal camouflage, it is essential to account for the emissivity changes with temperature. At increased temperatures, free-electron scattering becomes more prominent due to intensified electron–electron and electron–phonon interactions, causing an increase in the imaginary part of permittivity. Therefore, the absorption and emissivity of MXenes also rise with temperature, as indicated in Figure 8e.^10^

As temperature rises, thermal radiation follows a *T* ^4^ relationship according to the Stefan–Boltzmann law. Consequently, the relative contribution of radiative heat transfer compared to conduction increases markedly at higher temperatures, underscoring the importance of precise thermal radiation control for advanced thermal camouflage applications. A significant concern is the long-term thermal stability of MXenes under elevated temperatures, particularly due to their sensitivity to oxidation. As presented in Figure 9a, a Ti_3_C_2_T_*x*_ film exposed to 500 °C for more than 10 h exhibits outstanding thermal stability, with only slight variation in IR surface temperature.^29^ Nevertheless, in some other MXene systems, continuous exposure over 24 h may result in the gradual deterioration of thermal camouflage properties owing to oxidation-driven degradation. This challenge can be addressed by applying surface functionalization and the fabrication of MXene-polymer composite, which serves to suppress oxidation and improve long-term performance at high temperatures.

**Figure 9:**
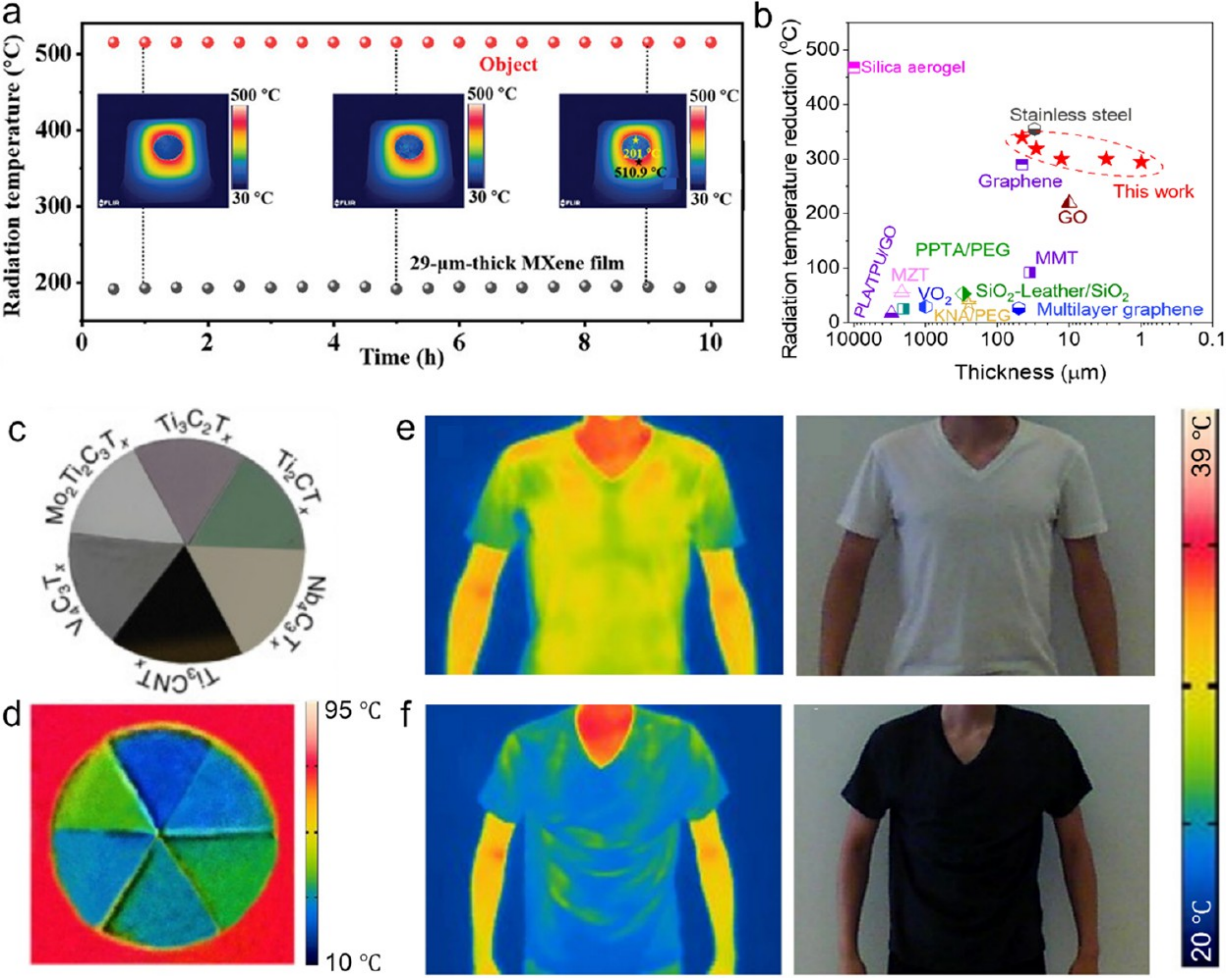
Demonstration of thermal camouflage performance utilizing MXenes. (a) Long-term high-temperature thermal camouflage stability achieved with MXene films. Reproduced with permission.^29^ Copyright 2021, John Wiley and Sons. (b) Comparison of IR surface temperature reduction for MXenes and conventional thermal camouflage materials across different film thicknesses. Reproduced with permission.^29^ Copyright 2021, John Wiley and Sons (c) Digital images displaying various MXene films at room temperature. (d) IR camera images of several MXene films situated on a hot plate maintained at 100 °C. Reproduced with permission.^10^ Copyright 2023, Elsevier. (e, f) Comparative demonstration of wearable thermal camouflage using (e) untreated T-shirts and (f) MXene-coated T-shirts. Reproduced with permission.^10^ Copyright 2023, Elsevier.

Figure 9b compares the thermal camouflage capability of MXenes with other reported thermal management materials.^29^ Although porous silica aerogels can achieve substantial IR temperature reduction due to their extremely low thermal conductivity, their function relies solely on conduction and demands thicknesses exceeding 10 mm. Furthermore, the limited mechanical integrity of aerogels restricts their practical utility. Conversely, Ti_3_C_2_T_*x*_ MXene films achieve efficient thermal camouflage at micron-scale thicknesses due to their low emissivity, removing the need for thick insulation layers. When compared with stainless steel and other 2D materials, MXene films exhibit equal or higher IR temperature reductions with much lower thickness. Notably, Ti_3_C_2_T_*x*_ MXenes synthesized from high-crystalline Al-rich Ti_3_AlC_2_ MAX phases offer further improvement, reaching electrical conductivities over 20,000 S cm^–1^ and an average IR emissivity of 0.06—at least 0.1 lower than the emissivity levels presented in Figure 9b (refer also to Figure 3).^29^

The primary objective of thermal camouflage is to match the thermal radiation profile to that of the surrounding environment, necessitating a broad and tunable emissivity spectrum. MXenes are highly suitable for this function because their emissivity can be adjusted from 0.06 to 0.49, enabling adaptation to a wide range of ambient scenarios as presented in Figure 9c,9d.^10^ By selecting MXene films with specific compositions, it is possible to finely control IR emission and engineer desired thermal signatures. For thermal camouflage at elevated temperatures, it is particularly important to preserve low emissivity to reduce thermal radiation contrast between the material and its background.

It is important to note that MXene coatings can be placed on any surface. We developed a universal salt-assisted assembly protocol for fast large-scale assembly of MXene coatings on polymer substrates.^73^ By adding NaCl or other salts to the MXene colloidal suspension in water, one can achieve substrate-independent MXene deposition. Coating high-performance polymers, such as Kevlar, with MXenes holds the potential for significant advancements in thermal management under extremely low and high temperatures, preventing heat loss and protecting equipment and personnel. However, coating every room in every building with a micron-thin MXene film can make a larger impact on energy saving and minimizing global warming than any other efforts underway. Figure 9e,9f illustrate the potential of MXene-coated fabrics for wearable thermal camouflage.^10^ A T-shirt coated with MXene using straightforward washing in a dilute colloidal solution of MXene in water shows IR surface temperatures that are approximately 10 °C lower than those of the uncoated T-shirt, even when exposed to the same body heat. This finding further demonstrates the advantageous solution-processability of MXenes and their compatibility with a wide range of substrates using deposition approaches such as dip-coating, spray-coating, and blade-coating for developing thermal camouflage systems.

Moreover, dynamic thermal camouflage is crucial for next-generation soft robotics, enabling adaptive concealment and real-time environmental responsiveness. Li et al. developed an interfacial engineering strategy to fabricate Ti_3_C_2_T_*x*_ MXene based soft robotic skin. The resulting MXene-based skin, featuring a tunable and reconfigurable microstructure, exhibits adjustable IR emissivity ranging from 0.3 to 0.8, thereby enabling dynamic thermal camouflage. Benefiting from the intrinsic Seebeck effect, controlled microcrack propagation, and excellent electrical conductivity, the MXene robotic skins seamlessly integrate thermal and strain sensing functionalities while simultaneously serving as deformable antennas for wireless communication. Without requiring additional electronic components, soft robots coated with conformal MXene skins autonomously achieve adaptive thermal camouflage through thermoelectric feedback in response to environmental temperature variations. Equipped with built-in strain sensing and wireless communication capabilities, these soft robots can record their locomotion paths and transmit critical information to subsequent units, facilitating coordinated movement and sustained concealment under thermographic surveillance.^31^

### 3.2 Camouflage Applications

#### 3.2.1 Infrared Anticounterfeiting

Infrared (IR) anticounterfeiting technologies are crucial for preventing fraud and verifying authenticity by incorporating hidden features that are undetectable to the naked eye but can be revealed through specialized IR imaging. Their significance lies in providing high security authentication that is difficult to replicate, as well as enabling dynamic or multifunctional verification in response to external stimuli such as specific IR wavelengths or temperature changes. These approaches are widely employed to protect intellectual property, ensure product authenticity in sectors such as pharmaceuticals, and safeguard high-value assets including banknotes and identification documents.^74^

Materials with tunable IR emissivity—invisible in the visible spectrum yet detectable under thermal or IR imaging—are particularly attractive for such applications. The IR emissivity and thermal camouflage performance of MXenes can be precisely controlled through surface functionalization using small or polymeric organic molecules. Notably, polydopamine (PDA) modification offers significant advantages in effectively passivating reactive terminations, forming a protective barrier that suppresses oxygen and moisture adsorption—thus preserving optical and electrical properties.

Figure 10a,b depicts the preparation process of polydopamine-modified Ti_3_C_2_T_*x*_ MXene (*p*-MXene) with adjustable IR emissivity.^30^ Dopamine (DA) monomer underwent prepolymerization for 2 h in a tris-buffer solution (pH ≈ 10) to prevent flocculation during subsequent grafting. The prepolymerized DA molecules were subsequently anchored to the MXene surface through both covalent and noncovalent interactions, yielding a uniform PDA nanolayer (Figure 10a).^30^ This functionalization strategy not only acts as a barrier against water and oxygen to enhance the oxidation resistance of MXene, but also improves the ink’s rheological behavior by increasing the dynamic volume at the molecular level. Moreover, by adjusting the content of PDA molecules, the infrared emissivity of the PDA nanolayer can be modulated (Figure 10b).^30^ Samples denoted as *p*-MXene-10 through *p*-MXene-50 correspond to MXene surfaces functionalized with PDA at varying loadings (10 to 50 wt.%). The unmodified MXene film displays a low IR emissivity of 19.6%, whereas pure PDA exhibits a high emissivity of 92.2%, attributed to its insulating properties. Increasing the PDA concentration progressively raises the emissivity of *p*-MXene from 0.21 to 0.37, as presented in Figure 10c.^30^ This trend results from the intrinsically high emissivity of PDA, modified surface chemistry, and the consequent decrease in electrical conductivity of *p*-MXene. Figure 10d presents thermal images of MXene and *p*-MXene films applied to a graphite plate (emissivity ∼ 0.90) at a substrate temperature of 70 °C. The measured apparent temperatures were 33.6 °C for MXene and 36.2, 37.6, 38.4, 42.6, and 47.4 °C for *p*-MXene-10, 20, 30, 40, and 50, respectively, all considerably lower than the graphite plate’s temperature (69.9 °C).^30^ Coatings with reduced emissivity emit less infrared energy, resulting in cooler observed temperatures despite identical substrate heating. The differences in infrared-detected temperatures among MXene, *p*-MXene coatings, and the graphite substrate are further detailed in Figure 10e, which demonstrates that the temperature gap increases with increasing PDA loadings and higher heating temperatures.^30^

**Figure 10:**
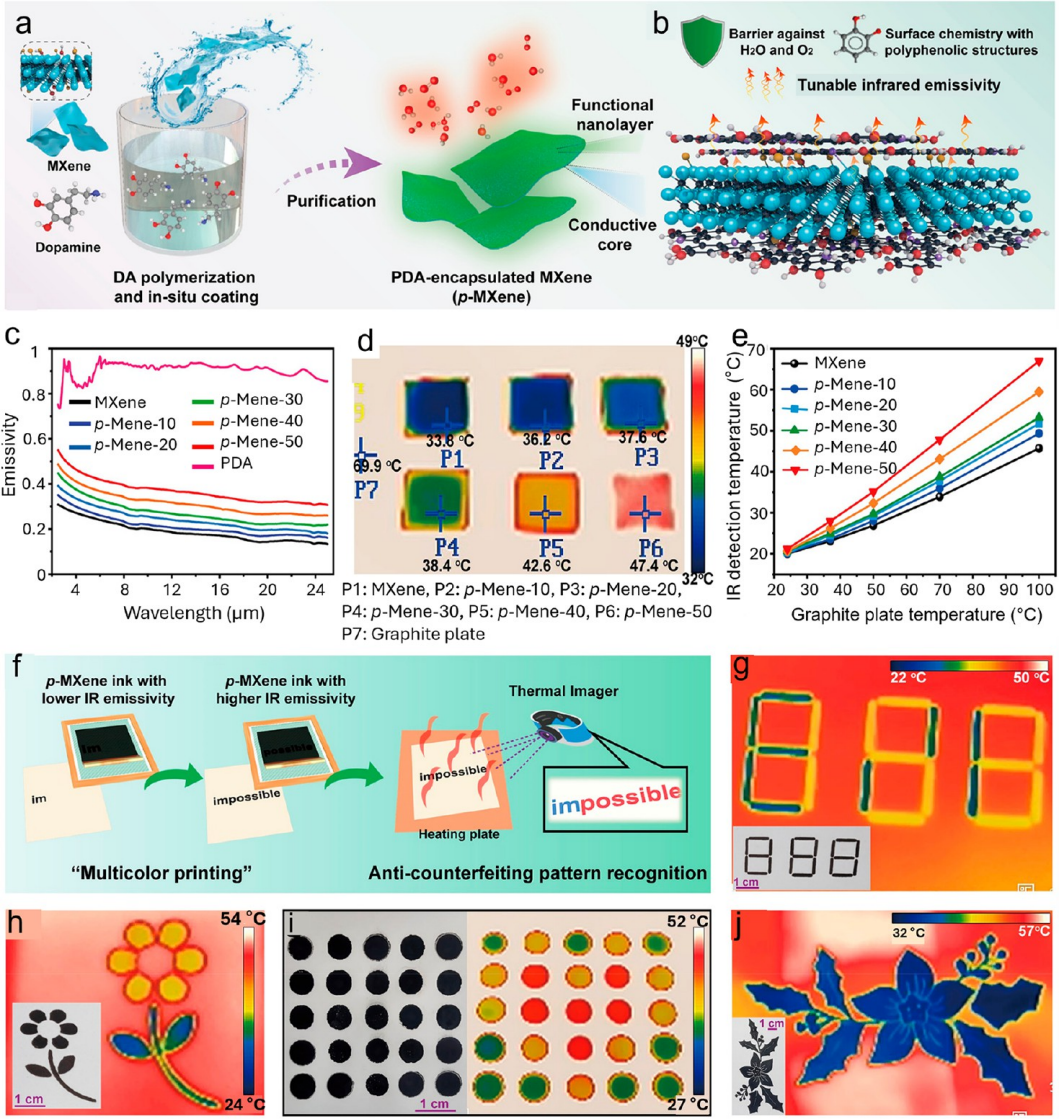
Influence of surface functionalization on the thermal camouflage performance of MXenes. (a) Schematic diagram illustrating the synthesis process of polydopamine-grafted MXene *(p*-MXene). (b) Illustration of the protection mechanism and adjustable infrared emissivity of *p*-MXene, emphasizing its ability to control thermal radiation. (c) Emissivity spectra comparison among MXene, *p*-MXene, and PDA across the mid-infrared band (2.5–25 μm). (d) Infrared thermal images showing MXene and *p*-MXene patterns on a graphite plate substrate at 70 °C. (e) The difference in infrared-detected temperature for MXene and *p*-MXene coatings relative to the graphite plate. (f) Screen-printing of infrared anticounterfeiting coating by using emissivity-adjustable *p*-MXene ink. Infrared thermal images of (g) cryptographic patterns, (h) security flower, and (i) security square matrix pattern demonstrating the concept of “multicolor printing”. (Insets: Digital images of security patterns). (j) IR camera image of complex printed flower with high-precision infrared anticounterfeiting. Reproduced with permission.^30^ Copyright 2022, American Chemical Society.

The *p*-MXene with tunable mid-infrared (mid-IR) emissivity presents an exciting opportunity for information security, encryption, and anticounterfeiting technologies. Their exceptional printability enables the creation of invisible yet thermally decodable security patterns. In particular, the controllable IR emissivity of printed *p*-MXene films allows for encoding information that remains indistinguishable under visible light yet can be distinctly recognized through infrared imaging. The *p*-MXene inks were employed to fabricate multilevel thermal images and encrypted patterns via screen printing (Figure 10f), Owing to the excellent rheology and surface adhesion of *p*-MXene dispersions, various complex patterns—including “multicolor” passwords, and flower motifs—were precisely printed onto flexible substrates such as paper (Figure 10g,h). These patterns appear visually identical in daylight but exhibit distinct emissive contrasts under a thermal imager when subjected to mild heating. Regions coated with low-emissivity MXene appear cooler (dark in the thermal image), while those with higher emissivity display warmer tones (bright regions). This temperature-dependent emissive contrast allows encoded information, such as hidden symbols or authentication marks, to be revealed only under specific thermal or IR conditions, achieving an efficient encryption–decryption mechanism.

By using three *p*-MXene inks with distinct emissivity values, an encrypted matrix capable of multilevel information encoding and advanced security protection was successfully fabricated (Figure 10i). Each MXene ink exhibited a precisely tailored mid-infrared emissivity, enabling the formation of a thermally responsive pattern where emissive contrast only appears under infrared observation. This emissivity modulation allows the encrypted matrix to reveal specific information sequences or authentication symbols when heated or imaged under controlled thermal conditions, establishing a hierarchical decoding system for enhanced information confidentiality and anticounterfeiting. Moreover, the superior rheological behavior and excellent printability of *p*-MXene inks permit high-resolution and precise printing of complicated and exquisite anticounterfeiting patterns as shown in Figure 10j. Such fine control over print morphology and emissivity contrast ensures that the patterns remain visually identical under ambient light but display clearly distinguishable temperature-dependent signatures in the infrared spectrum. This dual-mode invisibility—visible concealment and thermal visibility—offers exceptional potential for anticounterfeiting and authentication technologies.

Therefore, *p*-MXene inks with adjustable mid-IR emissivity provide a versatile platform for infrared information encryption, anticounterfeiting labeling, and anti-IR detection applications. By rationally designing the ink composition, concentration ratio, and printing architecture, both the emissive response and encoded information density can be customized for different levels of security. Additionally, the thermal deciphering process, which relies on a controlled heat source and thermal camera, enables straightforward yet secure decoding of hidden information. This approach not only strengthens the security level of MXene-based anticounterfeiting systems but also broadens their applicability to encrypted documents, identity tags, currency verification, and secure packaging technologies, ensuring robust protection against counterfeiting and unauthorized duplication.^30^

#### 3.2.2 Infrared Stealth for Defense and Security

Infrared (IR) stealth plays a vital role in reducing thermal detectability of military assets, including vehicles, drones, and personnel, by suppressing their infrared signatures to evade heat-seeking sensors and thermal imaging systems. Effective thermal camouflage and low-emissivity coatings are therefore essential to enhance survivability and operational secrecy in modern warfare, where IR-based surveillance and targeting technologies are increasingly dominant.^75^ In defense and security applications, materials are required to sustain their stealth functionality under extreme environmental conditions, demonstrating long-term durability and resistance to performance degradation. MXene composites provide notable benefits compared to pristine MXenes, especially in thermal camouflage applications. While unmodified MXene possesses low IR emissivity—making it effective for stealth and IR shielding—it may be prone to environmental degradation from oxidation and mechanical instability.^76^ To address these shortcomings, integrating MXene with a polymer matrix serves as a protective layer, reducing oxidation, strengthening mechanical properties, and extending service life.^77,78^

A representative example is provided by the study of Guo et al., who established a MXene-based composite system that was comodified with hyaluronic acid (HA) and hyperbranched polysiloxane (HSi). This dual modification approach contributed both to improved mechanical flexibility and to enhanced long-term IR stealth properties of the MXene composite.^79^ Although pristine Ti_3_C_2_T_*x*_ MXene demonstrates low infrared emissivity, incorporating it into a polymer composite containing HA, MXene, and HSi in a mass ratio of 3:3:2 (H_3_M_3_Si_2_) leads to a modest rise in emissivity (Figure 11a). Nevertheless, this compromise remains justifiable due to marked improvements in mechanical strength and environmental stability. A distinguishing attribute of the H_3_M_3_Si_2_ MXene composite is its outstanding flexibility. The composite film withstands repeated folding into intricate origami shapes such as cranes and frogs without exhibiting cracks or performance loss (Figure 11b). Additional durability is confirmed through prolonged thermal cycling assessments. After being subjected to 100 °C for as long as 50 days, the composite preserves stable surface resistance and reliable thermal radiation behavior, underscoring its capacity to resist cyclic thermal stress and mitigate environmental aging (Figure 11c). In the context of flexible composites, the interplay between IR emissivity and mechanical strength is critical for advancing thermal camouflage. According to Figure 11d, a sequentially densified MXene (SDM) film—produced by intercalating small Ti_3_C_2_T_*x*_ flakes and subsequently bridging them with Ca^2+^/borate ions—achieves an ultrahigh tensile strength of 739 MPa while sustaining a low emissivity of approximately 0.1.^80^ This densification procedure removes interlayer voids, promotes flake alignment, and reinforces interfacial cohesion, establishing a new standard for mechanically resilient, low-emissivity materials in IR camouflage applications.^81^

**Figure 11:**
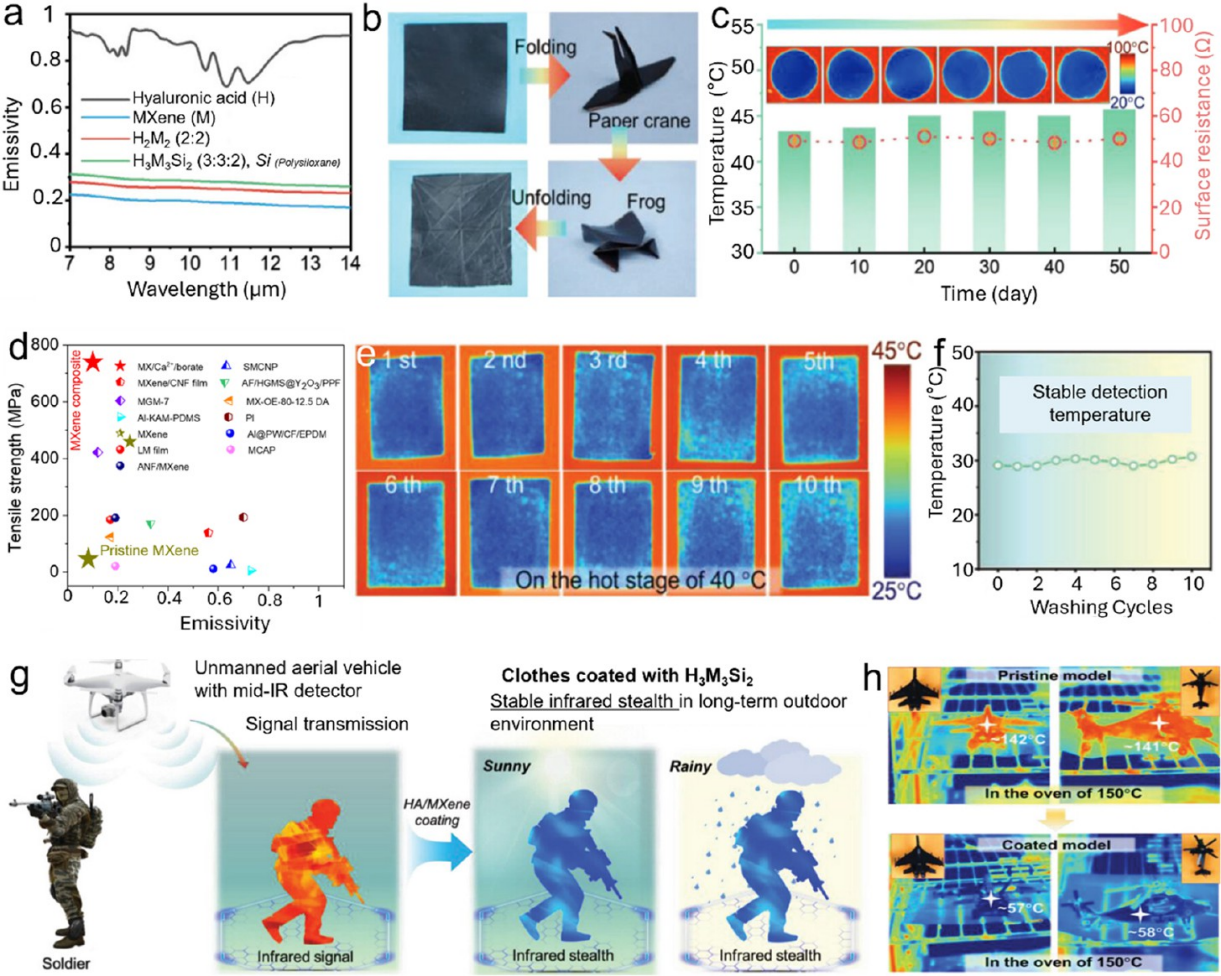
MXene composites for tunable thermal camouflage applications. (a) IR emissivity comparison between pristine Ti_3_C_2_T_*x*_ and its composites integrated with hyaluronic acid (HA) and hyperbranched polysiloxane (HSi), specifically H_2_M_2_ and H_3_M_3_Si_2_. H, M, and Si stand for HA, MXene, and HSi, respectively, and the values 2 and 3 indicate their respective mass ratios. (b) Images of MXene composite films subjected to bending and folding, demonstrating their enhanced flexibility and mechanical endurance. (c) Evolution of surface temperature and resistance in MXene composite films as a function of annealing duration at 100 °C, which demonstrates the sustained stability of their thermal radiation and electrical characteristics under repeated thermal cycling at elevated temperatures. Reproduced with permission.^79^ Copyright 2024, John Wiley and Sons (d) Tensile strength versus IR emissivity for various MXene and MXene composite films. The sequentially densified MXene (SDM) film—formed through intercalation of small flakes and Ca^2+^/borate ion bridging—is indicated by the red star symbol (739 MPa, 0.1 emissivity), surpassing most previous literature values.^80–82^ (e) Thermal infrared images of the H3M3Si2-coated textile before and after washing for different durations, and (f) the corresponding surface radiation temperature of the H3M3Si2 textile after various washing cycles. (g) Schematic of stable infrared stealth scenarios of the optimized H_3_M_3_Si_2_ composite coating onto cotton textile, showing promising prospect for the consistency of target and background for soldiers to avoid IR detection. (h) The thermal camouflage test of aircraft model coated with the H_3_M_3_Si_2_ composites, under high-temperature environments. Reproduced with permission.^79^ Copyright 2024, John Wiley and Sons.

The wash resistance of functional textiles plays a crucial role in determining their long-term practical applicability, particularly for wearable thermal camouflage and protection systems. To evaluate the durability of H_3_M_3_Si_2_ ink coated cotton textile, simulated machine-wash tests were conducted in dynamic water, where a magnet rotor stirred at 600 rpm for 10 min to mimic real washing conditions. Remarkably, even after multiple washing cycles, the coated textile maintained its structural integrity and stable surface performance. The thermal infrared images and corresponding surface radiation temperatures (Figure 6e,f) reveal that the H_3_M_3_Si_2_@cotton textile consistently retained a surface temperature of approximately 30 °C, regardless of the number of washing cycles. This minimal variation demonstrates the strong interfacial adhesion between the H_3_M_3_Si_2_ coating and the cotton substrate, as well as the chemical stability of the coating layer under mechanical agitation. The outstanding wash durability highlights the robustness and potential of H_3_M_3_Si_2_-coated textiles for long-term use in real-world environments such as personal thermal management, military uniforms, and stealth garments, where repeated washing and environmental exposure are unavoidable.

The excellent infrared stealth performance of the H_3_M_3_Si_2_ ink coated cotton textile is illustrated in Figure 11g. Before coating, the unmodified textile exhibits high infrared emissivity and poor thermal concealment, allowing the soldier body’s thermal radiation to be easily captured by infrared detectors or thermal imaging cameras, resulting in a bright, high-contrast silhouette against the background. After coating the textile with H_3_M_3_Si_2_/MXene composite ink, the surface becomes conductive, and more reflective in the mid-infrared range, significantly suppressing the outward emission of body heat. Consequently, the soldier wearing the H_3_M_3_Si_2_/MXene coated textile blends seamlessly with the surrounding environment, demonstrating a remarkable reduction in detectable thermal contrast and achieving effective thermal camouflage. The silane modification not only improves adhesion between MXene flakes and fabric fibers but also forms a hydrophobic and antioxidative protective layer that prevents oxidation and moisture-induced degradation of MXene, thereby maintaining low emissivity and stability under long-term operation. Owing to these characteristics, the coated fabric retains its stealth capability and mechanical integrity even in harsh environmental conditions such as rain, humidity, or prolonged sunlight exposure, ensuring reliable and durable performance for defense and outdoor applications.^79^

Moreover, the uncoated airplane models placed in an oven at 150 °C, the surface temperature of the pristine, uncoated models reached approximately 141–142 °C, exhibiting strong thermal radiation and appearing as bright regions under infrared observation (Figure 11h, top panel). While the models coated with H_3_M_3_Si_2_/MXene composite ink displayed a much lower apparent surface temperature of about 57–58 °C, indicating a substantial suppression of thermal emission and efficient heat insulation (Figure 11h, bottom panel). This remarkable decrease in surface radiation highlights the excellent infrared stealth performance of the H_3_M_3_Si_2_/MXene composite ink coatings and their suitability for high-temperature environments. These findings underline the potential of H_3_M_3_Si_2_/MXene composite ink coatings for next-generation infrared stealth applications in aerospace, defense, and thermal management systems.^79^

In addition to planar films, the design of material structures is pivotal in optimizing thermal management and camouflage performance. For example, a three-dimensional MXene/wood-plastic composite (WPC) foam serves as an efficient thermal insulator. The WPC-MXene foam is capable of masking heat signatures following 30 s of thermal exposure. This insulating effect is attributed to its aerogel-like architecture, which impedes heat flow and thereby facilitates passive thermal stealth.^82^

To achieve both low IR emissivity and high thermal insulation simultaneously, Dang et al. proposed a synergistic composite approach that incorporates an asymmetric structure based on aramid nanofiber (ANF)/MXene layers, comprising a dense compact top layer and a bottom aerogel layer with a 3D porous network.^83^ The upper ANF/MXene dense layer effectively suppresses radiative heat loss by providing low emissivity in the mid-IR (3–14 μm) range, whereas the underlying aerogel-like 3D porous ANF/MXene layer restricts conductive heat transfer and thus enhances thermal insulation. This bilayer architecture generates a synergistic effect, combining the benefits of emissivity regulation with improved thermal resistance. The integration of these functionalities leads to enhanced stealth performance against thermal imaging detection.

The asymmetric structure achieved the lowest recorded surface temperature of 76 °C when exposed to a constant background temperature of 100 °C, yielding a maximum temperature drop of 24 °C. This significant improvement exceeds the performance of both the dense and the porous configurations evaluated individually. The ANF/MXene dense film provides a surface temperature decrease of approximately 9 °C, attributed mainly to its low emissivity characteristic, while the aerogel-derived film delivers a larger ∼20 °C reduction through superior thermal insulation, albeit with higher emissivity. By integrating both mechanisms, the asymmetric hybrid delivered the most effective thermal camouflage.^83^ These results highlight the advantages of coupling emissivity modulation with thermal insulation in a single composite. MXene-based asymmetric composites—combining tunable emissivity, mechanical flexibility, and tailored porosity—provide a promising strategy for next-generation thermal camouflage applications.

## 4 Photothermal Properties and Applications of MXenes

### 4.1 Photothermal Conversion

Solar energy covers a wide spectral range, consisting mainly of UV, visible, and IR light, with each type possessing photons of energies within the electromagnetic spectrum. Photothermal conversion refers to the mechanism by which materials absorb these photons and transform their energy into heat. The creation of broadband photothermal materials that can absorb solar energy efficiently across this range is considered a critical approach for promoting energy sustainability. These materials show significant potential in diverse applications, such as solar desalination, photothermal therapy, flexible electronics, and catalysis.

As discussed in Section 3.1, different MXene compositions demonstrate tunable absorption in the UV–visible region, which arises from their Interband transition properties. These optical characteristics facilitate the optimization of solar absorption throughout the electromagnetic spectrum, especially within the 300–2500 nm range representative of the air mass 1.5 global (AM 1.5G) solar spectrum, ultimately increasing the efficiency of photothermal conversion. Additionally, the inherently low emissivity of MXenes assists in retaining the heat generated within the film, further enhancing their photothermal performance. Figure 12a shows the UV–vis–NIR absorption spectra for aqueous dispersions of various MXenes, including Ti_3_C_2_T_*x*_, Nb_2_CT_*x*_, V_2_CT_*x*_, TiNbCT_*x*_, TiVCT_*x*_, and NbVCT_*x*_.^84^ In comparison with conventional 2D nanomaterials, such as graphene oxide (GO),^85^ black phosphorus (BP),^86^ and tungsten disulfide (WS_2_),^87^ MXenes display an exceptionally broad absorption range, covering the UV to NIR regions. Among these MXenes, V_2_CT_*x*_ exhibits the most pronounced light absorption, while Ti_3_C_2_T_*x*_ possesses the weakest, a trend that corresponds to their photothermal response when subjected to 808 nm NIR laser irradiation (0.5 W cm^–2^), as illustrated in Figure 12b. Given that Ti_3_C_2_T_*x*_ is the most extensively studied MXene in existing literature, careful selection of MXene composition holds considerable promise for maximizing photothermal performance across a broad spectrum of technological applications. Figure 12c details the solar absorption and photothermal conversion efficiency achieved by various MXenes compared to benchmark materials.^6,88–114^ MXenes demonstrate nearly 100% photothermal conversion efficiency and solar absorption approaching 95%,^115^ underscoring their notable potential as leading-edge photothermal materials.

**Figure 12:**
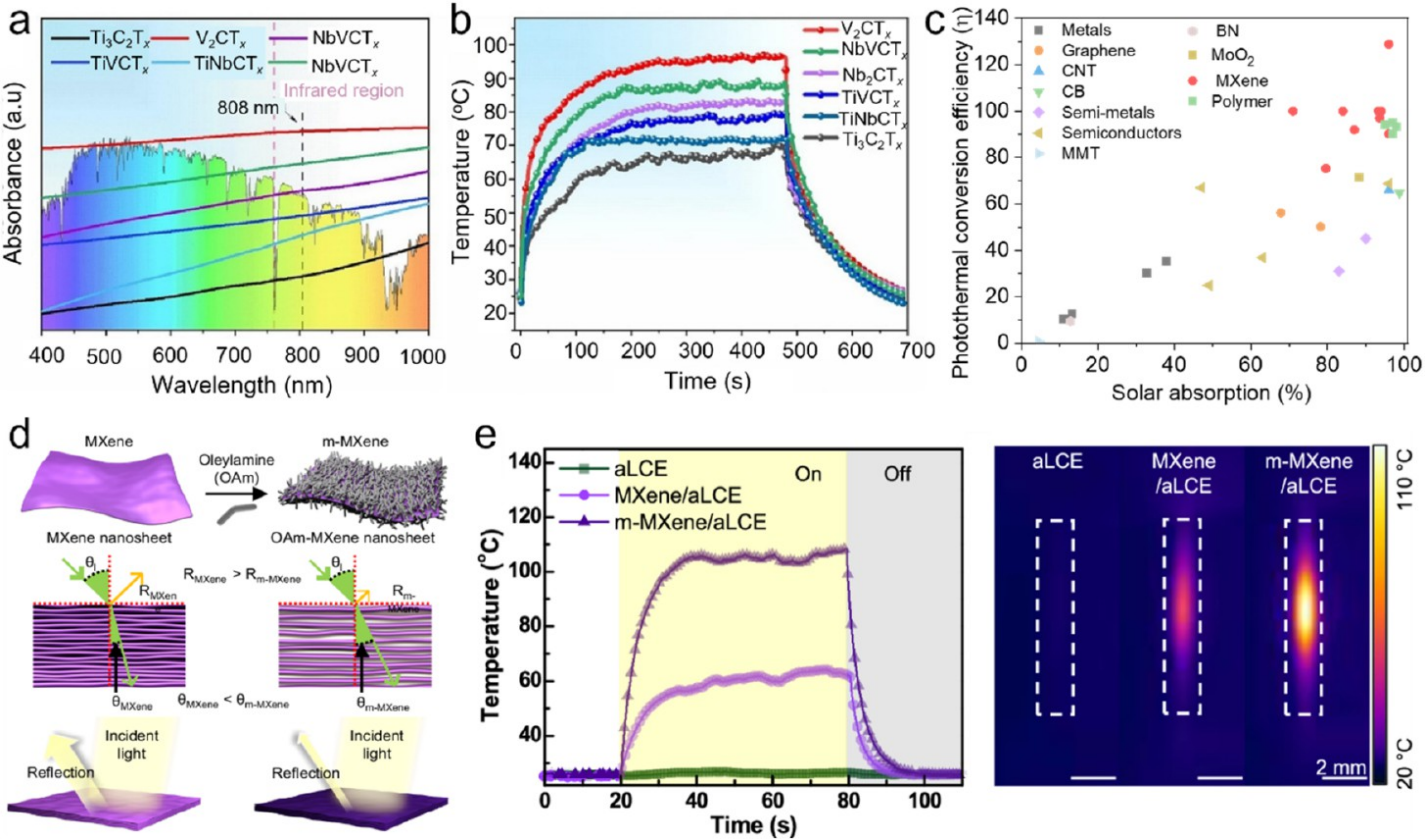
Photothermal conversion properties of MXenes. (a) UV–vis-NIR absorption spectra of Ti_3_C_2_T_*x*_, V_2_CT_*x*_, Nb_2_CT_*x*_, TiVCT_*x*_, TiNbCT_*x*_, and NbVCT_*x*_ MXenes. (b) Temperature profiles of these MXenes under 808 nm laser irradiation at a power density of 0.5 W cm^–2^. Reproduced with permission.^84^ Copyright 2023, Elsevier. (c) Comparison of solar absorption and photothermal conversion efficiencies among different materials, with MXenes denoted by the red symbol.^6,88–114^ (d) Schematic illustration of Ti_3_C_2_T_*x*_ MXene surface modification using an oleyl amine ligand and the associated change in incident light reflection; R and θ denote reflectance and angle, respectively. (e) Surface temperature profiles of aligned liquid crystal elastomer (aLCE), MXene/aLCE, and surface-modified MXene (m-MXene)/aLCE fibers under 808 nm laser irradiation. Reproduced with permission.^118^ Copyright 2022, Springer Nature.

To further improve the photothermal performance of MXenes, their surfaces are often functionalized with organic ligands or hybridized with metal oxides, carbon, or metal nanomaterials to promote plasmonic and photothermal effects and confer multifunctional characteristics.^116,117^ For example, Shin et al. successfully synthesized Ti_3_C_2_T_*x*_ MXene hybrids by attaching oleyl amine ligands to the MXene surface (Figure 12d).^118^ Alternating the assembly of MXene and organic layers led to a 2.6-fold decrease in light reflectance, resulting in photothermal conversion efficiency as high as 90%. When incorporated into aligned liquid crystal elastomer (aLCE) fibers, the surface-modified MXene (m-MXene)/aLCE fibers showed a 2.1-fold greater temperature increase than Ti_3_C_2_T_*x*_ MXene or unmodified aLCE fibers (Figure 12e). Moreover, the m-MXene/aLCE composite exhibited double the tensile strength (∼6.86 MPa) of aLCE, yielding significantly higher actuating stress (∼0.56 MPa) compared to MXene/aLCE (∼0.21 MPa). These results demonstrate not only an improved photothermal conversion attributed to altered properties of MXenes, but also the feasibility of simultaneously engineering other characteristics, such as mechanical strength, thereby expanding their potential across multiple application domains.

### 4.2 Photothermal Applications

#### 4.2.1 Solar Water Desalination

With increasing freshwater shortages driven by population growth and industrialization, solar desalination technologies that harness sunlight to remove dissolved salts from seawater have emerged as a highly promising approach. Interfacial solar steam generation utilizes photothermal materials positioned at the air/water interface to absorb solar radiation and convert it into localized heat, thereby driving the evaporation of seawater and enabling freshwater production.^119^ To enhance the efficiency of steam generation, materials with strong photothermal conversion properties, such as plasmonic metals,^120^ semiconductors,^121^ and carbon-based nanomaterials,^122^ are extensively used in solar-driven evaporation systems. Nevertheless, because solar desalination primarily takes place at the air–water interface, additional material characteristics are needed for optimal function: (i) low thermal conductivity to retain heat at the interface, (ii) the capacity to reduce radiative, convective, and conductive losses employing improved internal light reflection, and (iii) salt resistance to maintain long-term stability. In this context, MXenes have recently emerged as promising alternatives (Figure 13a). Their inherently low thermal conductivity confines heat at the evaporation surface, while their layered architecture supports multiple internal reflections, thereby minimizing losses. Moreover, MXenes possess favorable hydrophilicity and chloride salt-resistance, making them excellent candidates for interfacial solar desalination applications.

**Figure 13:**
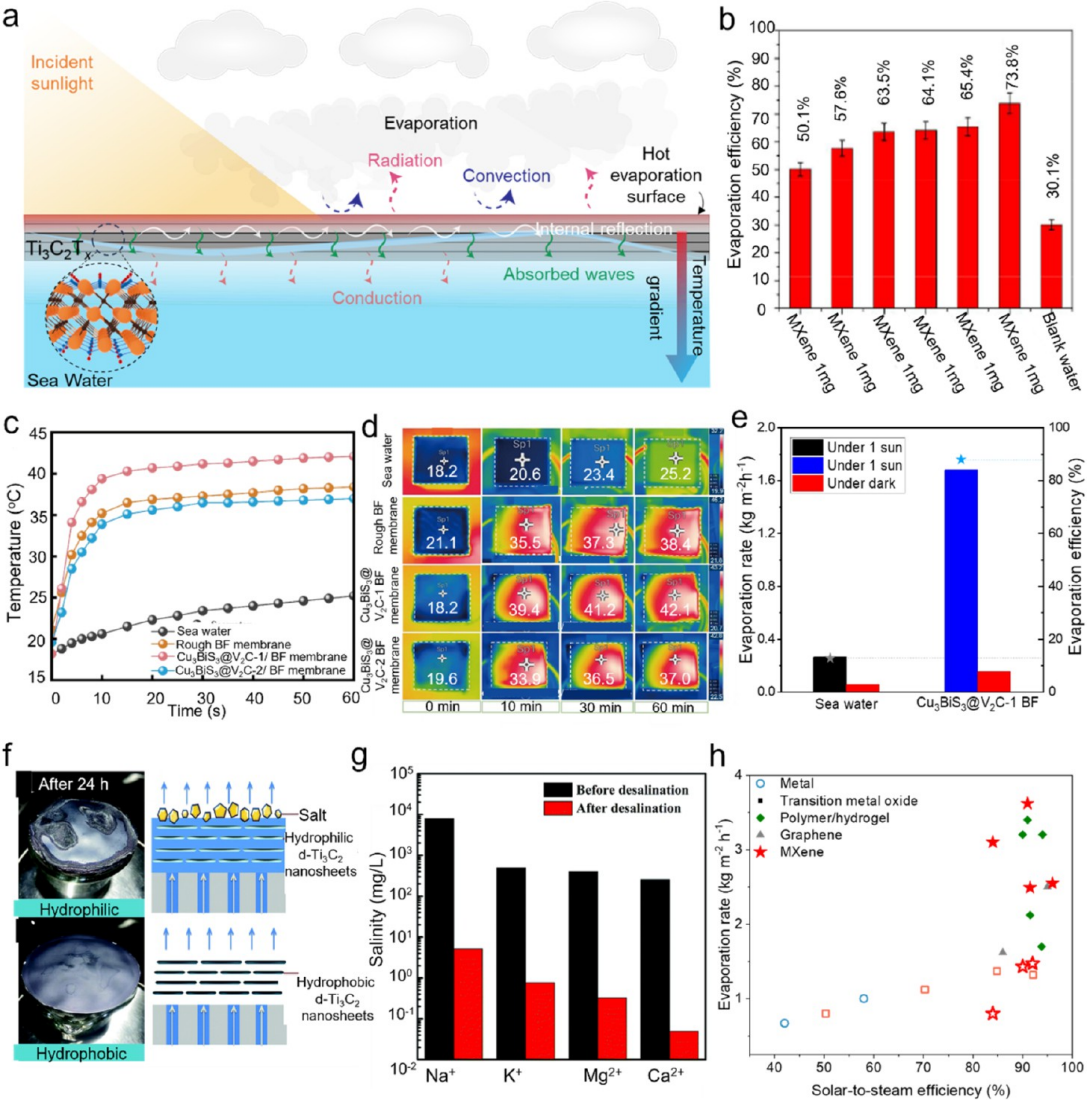
MXenes for solar water desalination. (a) Schematic illustration of factors affecting the solar desalination of MXenes. (b) Photothermal membrane evaporation efficiency as a function of Ti_3_C_2_T_*x*_ MXene mass loading. Reproduced with permission.^146^ Copyright 2017, American Chemical Society. (c) The surface temperature profiles and (d) thermal images of seawater, BF, Cu_3_BiS_3_/V_2_C-1/BF, and Cu_3_BiS_3_/V_2_C-2/BF membranes. (e) Evaporation rate and efficiency for seawater and Cu_3_BiS_3_@V_2_C-1/BF membrane. Reproduced with permission.^130^ Copyright 2022, Elsevier. (f) Surface-modified Ti_3_C_2_T_*x*_ nanosheets and schematic overview of the solar desalination mechanism for hydrophilic/hydrophobic membranes. (g) Effects on Na^+^, K^+^, Mg^2+^, and Ca^2+^ ion concentrations before and after desalination. Reproduced by permission.^28^ Copyright 2018, Royal Society of Chemistry. (h) Comparative evaluation of evaporation rates and solar-to-steam conversion efficiencies for MXenes and alternative materials.^132,134–145^

The pioneering study on the use of Ti_3_C_2_T_*x*_ MXenes for solar steam generation was conducted by Wang’s group.^27^ They found that the temperature of the MXene-poly­(vinylidene fluoride) (PVDF) membrane could reach up to 75 °C under 1 Sun illumination, which is significantly higher than the 30 °C observed for the bare PVDF substrate. The amount of evaporated water increased proportionally with Ti_3_C_2_T_*x*_ concentration, realizing an impressive 73.8% solar-to-water conversion efficiency at a 10 mg Ti_3_C_2_T_*x*_ loading under 1 Sun illumination (Figure 13b), a value that is competitive with leading materials employed in photothermal evaporation systems. In addition, MXenes have been incorporated into hydrogels, aerogels, and polymer composites to further improve solar desalination performance.^123–129^ The polymer matrices promote efficient water transport, decrease the evaporation enthalpy, and enhance antifouling performance, resulting in superior evaporation efficiency. For instance, Ti_3_C_2_T_*x*_-poly­(vinyl alcohol) (PVA) hydrogels have exhibited a notable solar evaporation efficiency of 128%,^124^ while Ti_3_C_2_T_*x*_-polyurethane composites incorporated into aerogels have reached evaporation rates up to 14.4 kg m^–2^ h^–1^.^125^ Furthermore, Ti_3_C_2_T_*x*_-coated polydimethylsiloxane (PDMS) fabrics have achieved an evaporation efficiency of 93.3% and an evaporation rate of 1.53 m^–2^ h^–1^,^126^ underscoring the exceptional promise of MXene-based materials in solar desalination technologies.

The application of V_2_CT_*x*_ MXene, recognized for exhibiting the highest photothermal effect among currently reported MXenes, has resulted in significant enhancements in solar desalination performance. In recent developments, bimetal sulfide/V_2_CT_*x*_ MXene-based photothermal nanocomposites have been synthesized for use in integrated solar steam generation, displaying exceptional outcomes in both desalination and wastewater purification.^130^ The Cu_3_BiS_3_@V_2_C/basalt fiber (BF) membrane displayed a rapid photothermal response, with its surface temperature swiftly increasing and stabilizing at 42.1 °C under solar irradiation (Figure 13c,d). This membrane demonstrated a high solar absorption rate of about 95.2% across the 300–2500 nm wavelength range and achieved an evaporation rate of 1.68 kg m^–2^ h^–1^ under 1-Sun illumination. The remarkable efficiency of the membrane is attributed to the broad-spectrum and efficient light absorption features of V_2_CT_*x*_ MXene, synergistic multilevel photothermal conversion provided by the composite architecture, and the reduction of water evaporation enthalpy via dual hydrogen-bonding interactions, resulting in a photothermal conversion efficiency of approximately 88.4% (Figure 13e).^130^

The hydrophobic characteristics of solar desalination membranes—an effective strategy to prevent salt crystallization and surface blockage can be efficiently achieved through MXene surface modification.^131,132,28^ For instance, Ti_3_C_2_T_*x*_ MXene was functionalized with trimethoxy­(1*H*,1*H*,2*H*,2*H*-perfluorodecyl)­silane (PFDTMS) and combined with polystyrene foam for thermal insulation and nonwoven fabrics to facilitate water transport (Figure 13f).^28^ PFDTMS undergoes hydrolysis followed by polycondensation, resulting in the formation of oligomers containing hydrophilic groups that interact with −OH groups present on the MXene surface. This process generates stable ether linkages and introduces −CF_3_ groups, thereby imparting hydrophobicity and improving desalination performance. The resulting membrane achieved a solar steam conversion efficiency of 71%, maintained structural integrity for 24 h, and provided over 99.5% salt rejection for typical seawater ions (Na^+^, K^+^, Mg^2+^ and Ca^2+^), in addition to near-complete removal of organic dyes and toxic metal ions such as Cu^2+^ and Cr^6+^ (Figure 13g). Beyond surface modification, hydrophobicity has also been introduced by incorporating a polymer layer onto MXene. Zhang et al. deposited polymeric nanospheres on the surface of Ti_3_C_2_T_*x*_ MXene using electrostatic spraying, producing superhydrophobic surfaces with enhanced desalination efficiency.^133^ Consequently, the hydrophobic MXene membranes demonstrated approximately 93.6% solar absorption, high freshwater generation of 2.88 kg m^–2^ h^–1^ and a solar-to-vapor conversion efficiency reaching 89%.

In conclusion, MXenes have established themselves as highly promising materials for solar desalination and water treatment, offering enhanced evaporation rates and superior solar-to-steam conversion efficiencies compared to other candidates (Figure 13h).^132,134–145^ Their distinctive photothermal characteristics, combined with the versatility of surface modification strategies, enable efficient water evaporation while mitigating salt accumulation and evaporation losses, making them optimal for advanced desalination applications.

#### 4.2.2 Photothermal Therapy

MXenes have gained recognition as promising materials for diverse biomedical applications, such as photothermal therapy (PTT), photoacoustic (PA) imaging, wound healing and drug delivery, owing to their exceptional in vivo biocompatibility, hydrophilicity, and efficient photothermal conversion. In cancer therapy, PTT provides a minimally invasive technique in which photothermal agents generate localized hyperthermia within tumor tissues. This targeted heating induces necrosis and/or apoptosis in cancer cells while preserving adjacent healthy tissues, thus reducing systemic adverse effects. The foundational study on photothermal tumor ablation utilizing Ti_3_C_2_T_*x*_ MXene was conducted by Lin et al.^27^ (Figure 14a). In this investigation, biocompatible Ti_3_C_2_T_*x*_ nanosheets demonstrated pronounced IR absorption and a photothermal conversion efficiency of 30.6% under 808 nm NIR laser irradiation. The Ti_3_C_2_T_*x*_ nanosheets functionalized with soybean phospholipid (Ti_3_C_2_T_*x*_–SP) displayed no notable toxicities in either in vitro or in vivo assessments. Additionally, intravenous administration of Ti_3_C_2_–SP or direct intratumoral injection of phase-changeable poly­(lactic-*co*-glycolic acid) (PLGA)/Ti_3_C_2_T_*x*_ implants resulted in effective tumor ablation upon NIR irradiation, with no recurrence observed. These findings underscore the strong potential of Ti_3_C_2_T_*x*_ MXenes as photothermal agents for cancer therapy.

**Figure 14:**
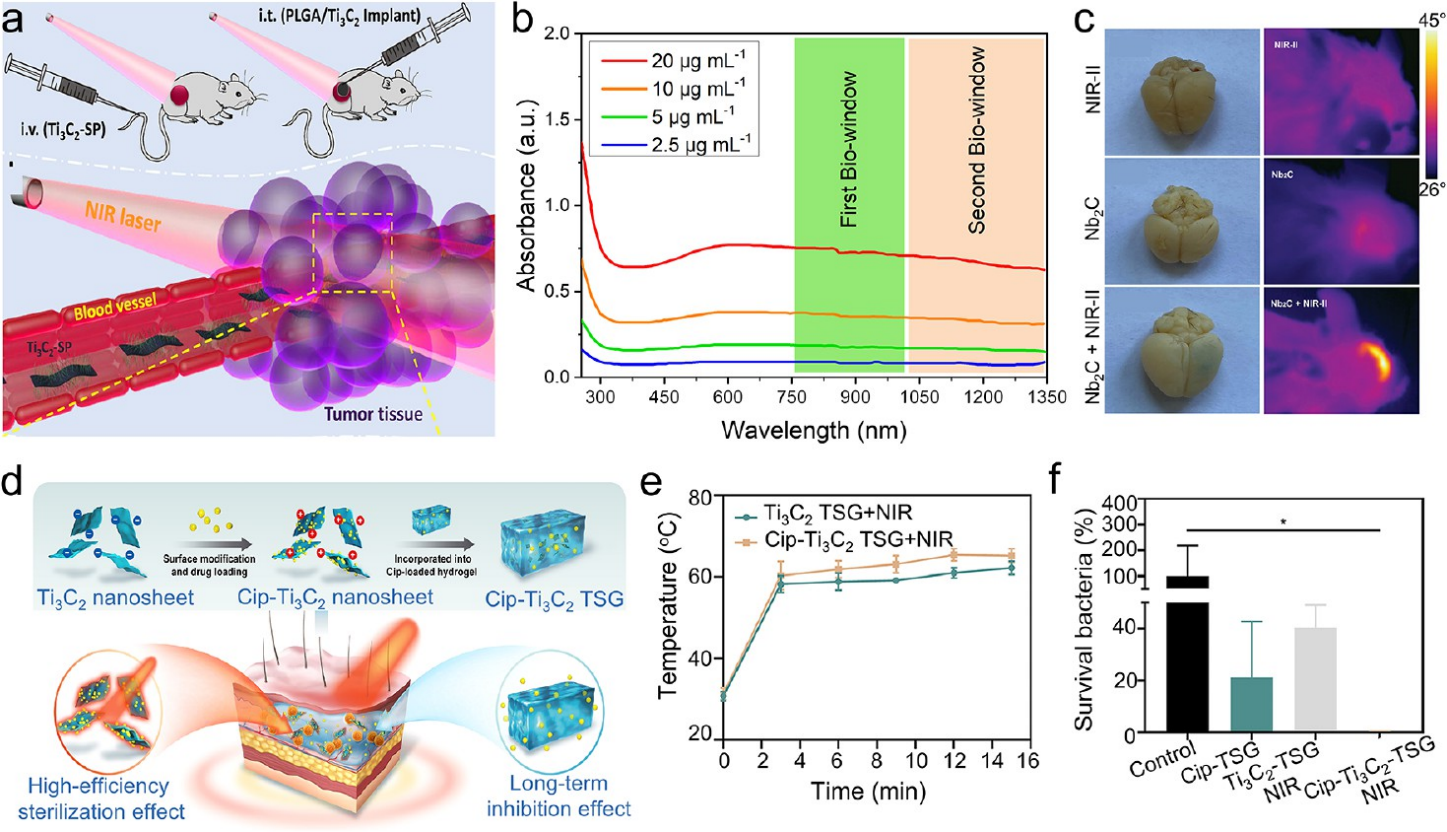
MXene in Photothermal Therapeutics. (a) Therapeutic strategies employing the photothermal capabilities of Ti_3_C_2_T_*x*_ nanosheets. Reproduced with permission.^27^ Copyright 2017, American Chemical Society. (b) Vis–NIR absorbance spectra of aqueous Nb_2_C dispersions at different concentrations. Inset: NIR-I (750–1000 nm) and NIR-II (1000–1350 nm). (c) Photograph of brains of C57BL/6 mice after photothermal therapy and IR Images of C57BL/6 mice under NIR-II laser irradiation. Reproduced with permission.^109^ Copyright 2017, American Chemical Society. (d) Schematic illustration of the antibacterial mechanisms of Cip- Ti_3_C_2_T_*x*_ nanocomposites used for dual-mode chemo-photothermal therapy. (e) Temperature variation curve during an in vitro antibacterial evaluation of Cip- Ti_3_C_2_T_*x*_ thermosensitive hydrogel (TSG) under NIR exposure. (f) Bacterial survival rate of methicillin-resistant *Staphylococcus aureus* (MRSA) after various treatments. Reproduced with permission..^153^ Copyright 2022, Elsevier.

Strong absorption in both the NIR-I (750–1000 nm) and NIR-II (1000–1350 nm) regions is essential for enhancing therapeutic outcomes, as longer wavelengths experience less tissue scattering and absorption, permitting deeper tissue penetration. Nb_2_CT_*x*_ MXene has been studied as a photothermal agent for theragnostic applications because of its robust absorption capability across the NIR-I and NIR-II regions (Figure 14b,c).^109^ The photothermal conversion efficiencies of Nb_2_CT_*x*_ MXene samples were reported to be between 34.9 and 36.4% under NIR-I irradiation, and further increased to 45.6% under NIR-II illumination, markedly surpassing conventional photothermal materials such as Au nanorods (21%), Cu_2–*x*_Se nanocrystals (NCs) (22%), and Cu_9_S_5_ NCs (25.7%).^109^ Motivated by its remarkable absorption and photothermal conversion efficiency in both NIR-I and NIR-II regions, photothermal therapy was conducted on mice using NIR-II lasers for noninvasive blood–brain barrier (BBB) disruption. Due to its outstanding photothermal performance, Nb_2_CT_*x*_ MXene elevated the local temperature in the mice’s brain to ∼43 °C, and the enhanced BBB permeability was confirmed by blue staining in the brain.^147^ In parallel, quantum dots of V_2_C MXene have been deployed for low-temperature photothermal therapy, with a high conversion efficiency of 45.04% in the NIR-II region, leading to total tumor cell ablation both in vitro and in vivo.^148^ Furthermore, MXenes can function as reduction substrates for the growth of metal nanostructures, promoting synergistic photothermal and chemical interactions between metals and MXene phases. He et al. reported that incorporating Pt nanoparticles onto V_2_C MXene significantly improved light absorption by nearly 100%. In a wound healing experiment, Pt@V_2_C used with NIR-II and H_2_O_2_ delivered notably better healing performance than other approaches, reaching a 99.2% wound area reduction after 10 days. Moreover, the Pt@V_2_C + H_2_O_2_ + NIR-II group exhibited a much lower bacterial load, underscoring its promise for combating deep-seated multidrug-resistant bacterial infections through combined photothermal and chemical effects.^149^

In addition, the diverse chemical compositions of MXene facilitate its utility in multifunctional photothermal therapy. For example, Ta_4_C_3_ MXene has been applied as a high-performance contrast agent for contrast-enhanced computed tomography (CT), while the incorporation of MnO_*x*_ enables tumor microenvironment-responsive imaging contrast.^113^ Following intravenous injection of MnO_*x*_/Ta_4_C_3_T_*x*_ into tumor-bearing mice, X-ray computed tomography imaging demonstrated a marked increase in tumor signal intensity, with Hounsfield units (HU) rising from 78 HU preinjection to 132 HU postinjection. This prominent enhancement is linked to the presence of Ta, an element with a high atomic number (*Z* = 73) and significant X-ray attenuation coefficient (4.3 cm^2^ kg^–1^ for Ta and 5.16 cm^2^ kg^–1^ for Au at 100 eV), emphasizing its suitability as a CT contrast agent.

MXene-based composites and hybrids have attracted considerable interest for dual-mode cancer imaging and photothermal therapy owing to their strong NIR absorption characteristics. As one example, Pt nanoparticles-functionalized Ti_3_C_2_T_*x*_ MXenes with polyethylene glycol modification demonstrated a high extinction coefficient (α = 4.81 L g^–1^ cm^–1^) at 1064 nm and an elevated photothermal conversion efficiency of 31.78%.^150^ Moreover, MXene-based hydrogels, including a cellulose-based Ti_3_C_2_T_*x*_ hydrogel loaded with doxorubicin hydrochloride, were investigated for their capability in combined photothermal and chemotherapy.^151^ Additionally, Ti_3_C_2_T_*x*_-PVA hydrogels exhibited remarkable antibacterial efficacy, eliminating up to 91.25% of bacteria and promoting 95.39% wound healing within 12 days,^152^ demonstrating their promise for use in photothermal therapy applications.

Additionally, MXenes have demonstrated significant potential as carriers for the loading, delivery, and controlled release of diverse therapeutic agents, including small-molecule anticancer drugs, enzymes, and therapeutic genes. For instance, a Cip-Ti_3_C_2_T_*x*_ hybrid hydrogel was fabricated by incorporating Ti_3_C_2_T_*x*_ MXene with the antibiotic ciprofloxacin (Cip) for combined chemo-photothermal therapy (Figure 14d).^153^ This thermosensitive hydrogel experiences a sol-to-gel phase transition at body temperature, which allows for localized drug delivery. When subjected to 808 nm NIR laser irradiation, the system achieved effective photothermal conversion accompanied by a pronounced temperature increase, promoting enhanced drug release. The Cip- Ti_3_C_2_T_*x*_ hydrogel achieved bactericidal efficiency exceeding 99.99999% against methicillin-resistant *S. aureus* (MRSA), enabling synergistic antibacterial action through the combination of photothermal therapy and chemotherapy. In in vivo studies, a notable temperature rise (∼60 °C) at the infection sites was observed and sustained for 12 min, supporting the in vivo photothermal conversion capability of the hydrogel (Figure 14e). The hydrogel system exhibited strong antibacterial efficacy in an MRSA-induced murine abscess model, reducing bacterial load by 99.6% and preventing infection relapse following NIR treatment (Figure 14f).^153^ Collectively, these findings underscore the promise of the Cip- Ti_3_C_2_T_*x*_ hybrid hydrogel for efficient antibacterial therapy and NIR-triggered targeted drug delivery.

#### 4.2.3 Other Applications

The remarkable photothermal characteristics of MXenes extend their practical scope far beyond water desalination and conventional photothermal therapy, enabling advancements in wearable electronics, self-healing materials, sensors, and energy devices such as microbial fuel cells (MFCs). For example, a hydrogel composed of poly­(*N*-isopropylacrylamide) and Ti_3_C_2_T_*x*_ displayed exceptional photothermal responsiveness and was implemented in a light-activated microfluidic system.^154^ A composite comprised of silver nanoparticles (AgNP)@Ti_3_C_2_T_*x*_ MXene and polyurethane (PU) exhibited efficient self-healing, restoring structural integrity within 5 min under light irradiation with a healing rate of 98%.^155^ Additionally, Chao et al. constructed a flexible and breathable sensor by integrating MXene nanosheets and silver nanowires (AgNWs) onto an electrospun thermoplastic polyurethane (TPU) nanofiber network coated with chitosan (CS), resulting in excellent sensing and photothermal heating performance.^156^

Similarly, MXenes have been utilized in MFCs, which are highly sensitive to temperature fluctuations that influence microbial bioelectrocatalytic activity. MFCs typically function optimally at approximately 30 °C, necessitating the use of external heating sources. Wang et al. fabricated a solar photothermal electrode incorporating Ti_3_C_2_T_*x*_ MXene to efficiently capture microbial energy at low ambient temperatures, thereby eliminating the need for bulk water heating.^157^ Leveraging the light-to-heat conversion capability of Ti_3_C_2_T_*x*_ MXene, the temperature of a carbon felt/MXene electrode immersed in cold water increased from 20.3 to 32.3 °C under 1 Sun irradiation. This led to a marked enhancement in current densities, reaching approximately 1.25 mA cm^–2^, confirming the effective harvesting of microbial energy in low-temperature conditions without necessitating heating of the entire water volume.

In summary, MXenes possess outstanding photothermal energy conversion efficiency across the UV–vis-NIR range, stemming from their bandgap energies and Interband transition properties, and retain the generated heat due to their low thermal conductivity and emissivity. These attributes enable their application across a wide spectrum of photothermal energy-driven applications, including solar desalination, photothermal therapeutics, wearable electronics, and microbial fuel cells. Moreover, their diverse chemical compositions and tunable surface chemistries provide additional versatility and multifunctionality across these platforms, offering clear advantages over conventional photothermal materials.

## 5 Electrothermal Properties and Applications of MXenes

### 5.1 Electrothermal Conversion Properties

Since the mid-19th century, when British physicist James Prescott Joule first described the Joule effect, the Joule heating phenomenon has been recognized as a fundamental thermodynamic principle, explaining the conversion of electrical energy into heat and leaving a lasting impact on both industrial production and scientific research.^158^ Joule heating materials are now widely employed in thermal management technologies, including energy-efficient heating and cooling, personal thermal regulation, automotive defrosting, and thermotherapy.^159,160^ Metals have traditionally been the materials of choice due to their high electrical conductivity (∼10^7^ S m^–1^) and ready commercial availability. However, their high density (>7 g cm^–3^), low optical transparency, poor flexibility, and susceptibility to surface oxidation constrain their practical utility.^161^ Modern electrothermal heating technologies demand materials that combine low density, optical transparency, mechanical flexibility and robustness, long-term stability, and facile processability.^162,163^

Low-dimensional carbon-based materials such as carbon nanotube and graphene show promise for Joule heating applications due to their high electrical conductivity even at low densities (<2.2 g cm^–3^) and favorable optical properties.^164,165^ However, their poor processability limits large-scale integration. Reduced graphene oxide (rGO) offers improved solution processability, enabled by polar surface groups,^166–168^ but suffers from reduced electrical conductivity due to lattice disruption during topotactic surface enrichment,^169^ as well as sustainability concerns arising from the complex reduction process and use of toxic chemicals.^170^

Recently, MXenes have emerged as promising Joule heating materials, combining exceptional metal-like conductivity (10^6^ S m^–1^) with pronounced anisotropic thermal conductivity—an efficient in-plane thermal conduction network with lower out-of-plane conductivity—making them particularly suited for Joule heating. Their hydrophilic surface terminations (−OH, −F, =O) and facile surface functionalization enable excellent dispersibility in polar media (e.g., water or polar organic solvents, such as isopropanol, propylene carbonate, etc.),^171^ positioning MXene-based heaters as a versatile alternative to other 2D materials. Moreover, their combination of metallic and ceramic characteristics imparts high fire resistance,^172^ enhancing their potential for durable and efficient electro-to-thermal energy conversion technologies.

Park et al.^25^ first reported the fabrication of transparent MXene-based thin film heaters (TFHs) on a glass substrate via spin coating, achieving remarkable optical transmittance (>65%) together with low sheet resistance (215 Ω sq^–1^). At a concentration of 6 mg mL^–1^, the MXene TFH maintained high transparency and exhibited a uniform temperature profile during operation, as confirmed by the homogeneous color distribution in pseudocolor IR images. Notably, no localized heat loss was observed along the contact metal, attributed to the highly uniform dispersion of MXene flakes on the glass surface (Figure 15a). Furthermore, Ti_3_C_2_T_*x*_ TFHs exhibited superior properties, attaining lower sheet resistance at comparable transmittance relative to graphene-based thin films prepared through similar processes (Figure 15b).^24^

**Figure 15:**
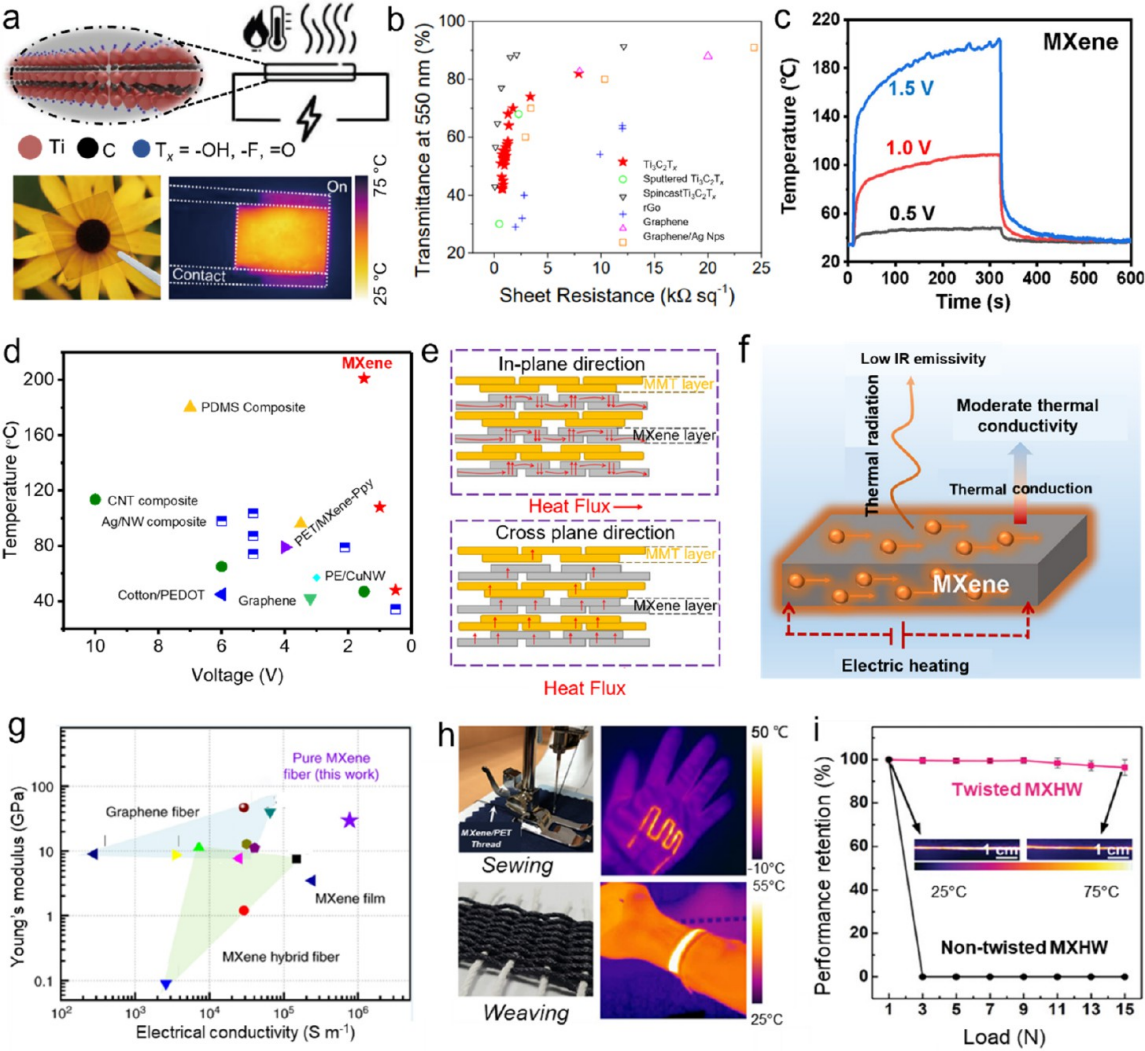
Joule heating with MXenes. (a) Schematic representation of Joule heating in Ti_3_C_2_T_*x*_ MXene, along with a photograph of the transparent MXene thin film heater (TFH) on glass prepared from a 6 mg/mL MXene dispersion, and corresponding IR images of the MXene TFH operated at 10 V. Reproduced with permission.^19^ Copyright 2019, American Chemical Society. (b) Comparison of optoelectronic properties between Ti_3_C_2_T_*x*_ thin films and other carbon-based films. Reproduced with permission.^24^ Copyright 2016, John Wiley and Sons. (c) Surface temperature profiles of MXene films at varying driving voltages, as measured by an infrared camera. (d) Comparative analysis of electric heating performance among various electric heaters. Reproduced with permission.^17^ Copyright 2023, Elsevier. (e) Schematic representation of anisotropic thermal conduction in the MXene/MMT composite films. Reproduced with permission.^175^ Copyright 2019, American Chemical Society. (f) Schematic of the electrothermal energy conversion process and heat loss pathways in MXene-based Joule heaters. Reproduced with permission.^17^ Copyright 2023, Elsevier. (g) Ashby plot showing the relationship between electrical conductivity and Young’s modulus of MXene fibers compared with other materials. Reproduced with permission.^180^ Copyright 2020, Springer Nature. (h) Fabrication of MXene-based heaters using MXene threads via sewing and weaving techniques. Reproduced with permission.^19^ Copyright 2019, American Chemical Society. (i) Assessment of the performance retention of the MXene heating wire (MXHW) heater subjected to different levels of tension. Reproduced with permission.^181^ Copyright 2024, John Wiley and Sons.

The performance of an electrothermal heater is determined not only by its electrical-to-thermal energy conversion efficiency but also by the extent of heat loss to the environment.^173^ Despite this, heat dissipation in electric heaters is often overlooked. The primary loss mechanism is thermal radiation,^174^ governed by Stefan–Boltzmann’s law, which dictates that radiative heat loss is directly proportional to the material’s IR emissivity (ε). Thus, reducing IR emissivity is crucial for minimizing radiation losses and maximizing electrothermal efficiency.

Shen et al. examined the electrothermal performance of MXene films by varying the input voltage using a DC power supply and monitoring temperature changes using both an IR camera and a digital camera.^17^ MXene films exhibited a rapid thermal response, with surface temperature increasing sharply upon voltage application. Even at a low voltage of 0.5 V, the films reached a saturated surface temperature of 48 °C, while voltages of 1 and 1.5 V produced temperatures of 108 and 201 °C, respectively, all within 25 s (Figure 15c). Due to the inherently low IR emissivity of MXene, the IR camera readings were lower, measuring 28.9 °C, 44.5 °C, and 75.5 °C for 0.5, 1.0, and 1.5 V, respectively. Notably, the saturated heating temperatures achieved at such low driving voltages rank among the best reported for electric heaters (Figure 15d), underscoring MXene’s exceptional Joule heating efficiency with minimal energy consumption.

Beyond high electrical conductivity, MXenes possess intrinsically low thermal conductivity and IR emissivity, offering distinct advantages over other 2D materials such as graphene in electrothermal applications. To further enhance thermal management characteristics, Li et al. incorporated 10 wt.% montmorillonite (MMT) into MXene films, producing an anisotropic thermal conductivity profile—high in-plane direction coupled with low out-of-plane conductivity—which enhanced directional heat dissipation.^175^ The resulting MXene/MMT composite film exhibited a nacre-inspired layered nanostructure, with MMT sheets intercalated into MXene sheets, promoting highly ordered in-plane alignment. Hydrogen bonding between MMT and MXene (Figure 15e) increased interflake contact area, reducing interfacial thermal resistance and expanding conductive pathways for planar heat transfer.^176^ Consequently, the outstanding Joule heating performance of MXene-based films can be attributed to their high electrical conductivity combined with minimized heat loss through both radiation and conduction (Figure 15f).

Two-dimensional (2D) materials offer variety of benefits—including facile assembly, high flexibility, and remarkable physicochemical characteristics—positioning them as promising building blocks for wearable electronics and flexible devices.^177,178^ Particularly, fiber-shaped conductors derived from 2D materials have garnered substantial attention for their lightweight, flexibility, and textile compatibility, enabling seamless integration into smart fabric systems.^179^

However, for MXenes, conventional fiber fabrication techniques—such as MXene/polymer blending or coassembly with reduced graphene oxide (rGO)—often reduce inherent conductivity due to the inclusion of insulating components. Moreover, the weak van der Waals interactions between small MXene flakes hinder self-supporting 1D fiber formation, and the typically low concentrations of MXene dispersions complicate direct fiber spinning.

To address these limitations, Eom et al. developed a binder-free, composite-free wet-spinning strategy to produce MXene fibers with superior electrical conductivity.^180^ When the dispersion concentration reaches ≥15 mg mL^–1^, Ti_3_C_2_T_*x*_ MXene form a stable liquid-crystalline phase with high viscosity, free of aggregation. At 25 mg mL^–1^, the dispersion behaves as a viscous ink (3.87 × 10^3^ Pa·s) and exhibits birefringence under crossed polarizers, confirming shear-induced molecular alignment. Successful fiber spinning is achieved when the storage-to-loss modulus ratio (*G*′/*G*″) lies between 1.80 and 6.36. For instance, at a low concentration of 5 mg mL^–1^ (*G*′/*G*″ = 13.33), fiber formation fails due to insufficient structural strength, whereas concentrations ≥15 mg mL^–1^ (*G*′/*G*″ = 5.29) enable continuous fiber production. The liquid-crystal MXene dispersions are extruded into a coagulation bath containing NH_4_^+^ ions, which induce gelation, yielding meter-scale, axially aligned MXene fibers with a dense lamellar architecture. The resulting MXene fibers exhibited an electrical conductivity of 7713 S cm^–1^ (Figure 15g), far surpassing that of conventional spun fibers. The Ashby plot analysis revealed that this electrical conductivity is 107 times greater than MXene/graphene hybrids, 27 times higher than MXene/PEDOT:PSS fibers and exceeds that of conventional graphene fibers by a factor of 12–220. This combination of outstanding conductivity and mechanical strength positions MXene fibers as highly promising candidates for wearable Joule heating devices.

Beyond wet-spun fibers, MXene has also been directly deposited onto polymeric fibers for use in wearable electrothermal heating systems. Park et al. demonstrated Ti_3_C_2_T_*x*_-coated poly­(ethylene terephthalate) (PET) fibers and textiles, producing flexible and efficient Joule heaters.^19^ Incorporated into gloves and fabrics, these MXene threads enabled localized heating up to 55 °C at 2.0–3.3 V cm^–1^ (Figure 15h), with rapid thermal response and precise spatial control, confirming their potential for dynamic wearable heating applications.

To enhance mechanical durability and long-term reliability, Jeong et al. proposed a biomimetic coassembly strategy inspired by the spiral grip of morning glory vines, creating highly robust MXene heating wires (MXHW) and fabrics.^181^ In this design, Ti_3_C_2_T_*x*_ MXene/polyacrylonitrile (PAN) composite fibers were helically wrapped around a nylon thread core and insulated with a poly­(vinylidene fluoride) (PVDF) coating. Surface functionalization of Ti_3_C_2_T_*x*_ via diazonium chemistry using 4-aminobenzoic acid improved mechanical cohesion and interfacial bonding. The fabricated MXHW maintained stable electrothermal heating performance under repeated elongations (up to 17.5%), withstood 12,000 bending cycles, and retained environmental stability at 99% relative humidity and 80 °C. The spiral structure (Figure 15i) produced elongated filaments that significantly enhanced mechanical robustness. Under a tensile load of 15 N, the MXHWs preserved approximately 98% of their original heating efficiency without failure. This design offers a durable, adaptable, and environmentally stable wearable heating platform, enabling seamless integration into smart textiles and future flexible electronics.

### 5.2 Electrothermal Applications

#### 5.2.1 Electrothermal Therapy

MXene-based Joule heaters hold significant potential for electrothermal therapy, particularly in treating bacterial infections. Pathogenic bacteria, such as *S. aureus*, can cause severe inflammation and even life-threatening complications. Although antibiotics remain the standard treatment, the rise of antibiotic resistance has prompted the search for alternative strategies. Existing antibacterial approaches using metal ions, polymers, or nanomaterials have shown efficacy but often leave toxic residues that hinder wound healing.

Wearable wound dressings with integrated Joule heating offer a noninvasive and residue-free therapeutic option. Zhao et al. developed an MXene-based smart fabric (M-fabric) by depositing 2D Ti_3_C_2_T_*x*_ MXene nanosheets onto cellulose fiber nonwoven fabric through strong MXene–cellulose interactions. This M-fabric serves as a wearable dressing capable of providing Joule heating-assisted antibacterial action and promoting wound healing.^182^ In a *S. aureus*-infected wound model (Figure 16a), the nonheated M-fabric reduced bacterial counts by ∼30%, attributed to MXene’s membrane-disruptive properties. Upon Joule heating, the fabric rapidly reached temperatures above 50 °C, efficiently eradicating bacteria and substantially enhancing wound healing rates. After 12 days, wounds treated with Joule-heated M-fabric achieved near-complete closure, while untreated and nonheated M-fabric groups retained 20 and 15% wound areas, respectively.

**Figure 16:**
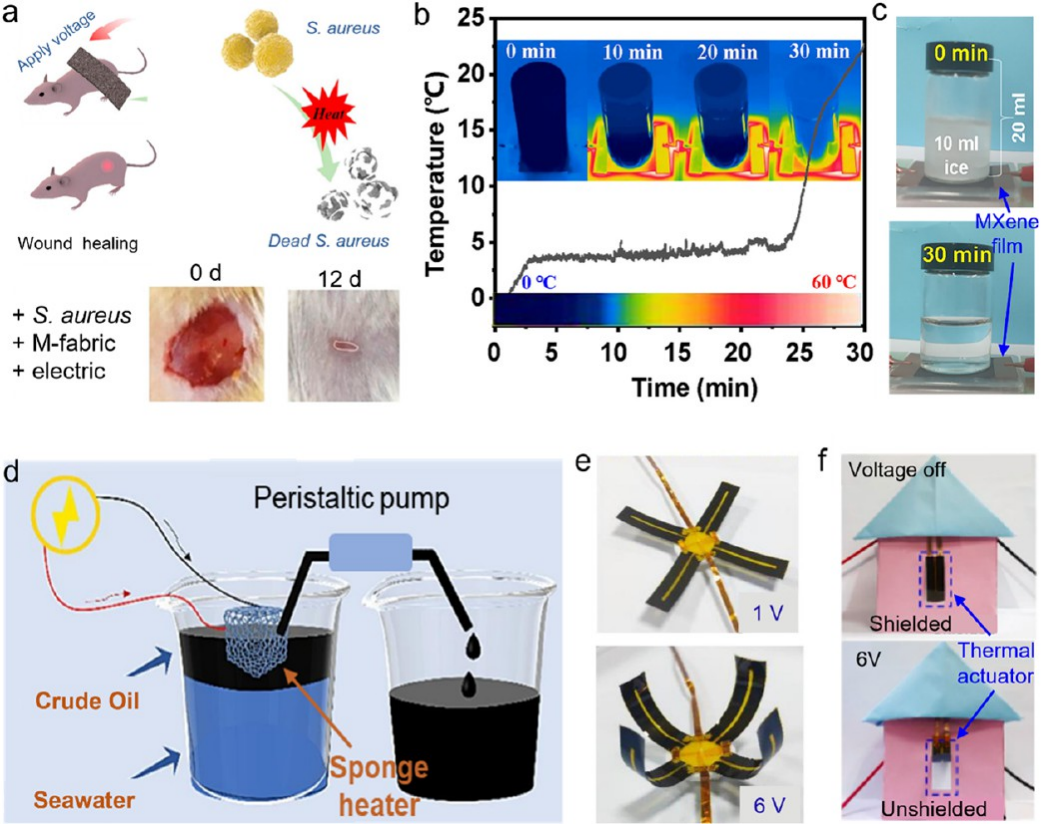
Joule heating applications (a) Schematic representation of electrothermal therapy applied to bacterial-infected wounds, illustrating the inactivation of *S. aureus* using an MXene-based Joule heater. Reproduced with permission.^182^ Copyright 2020, American Chemical Society. (b) Temperature–time evolution at the bottom of an ice/water glass cup (10 cm^3^) during deicing via an MXene film Joule heater operated at 1.5 V. Insets present IR thermal images captured at different stages of the deicing process. (c) Corresponding photographs showing the ice/water glass cup before and after deicing for 30 min. Reproduced with permission.^17^ Copyright 2023, Elsevier. (d) Schematic depiction of a vacuum-assisted crude oil recovery process utilizing electrothermal heating. Reproduced with permission.^183^ Copyright 2024, Elsevier. (e) Photographs display a four-finger PTFE/MXene/PI actuator undergoing thermal deformation into a flower-like configuration at applied voltages of 1 and 6 V, respectively. (f) Photographs illustrate the practical application of the MXene-based thermal actuator as a responsive window curtain. Reproduced with permission.^184^ Copyright 2024, Elsevier.

Additionally, the M-fabric showed humidity-responsive, reversible structural changes caused by water-driven swelling and contraction within MXene interlayers. This behavior supports real-time respiration monitoring and integrated temperature safety alarms by yielding detectable signals as water evaporates during heating, thereby enhancing thermotherapy safety and reducing the risk of burns.

### 5.3 Deicing

MXene-based Joule heaters are also highly promising for deicing applications. Ice accumulation poses serious challenge for aviation, transportation, and infrastructure in cold climates. Shen et al. demonstrated a MXene/graphene film deicing device (3 × 3 cm^2^) powered by a low-voltage DC source (Figure 16b).^17^ At 1.5 V, a 10 cm^3^ ice block placed atop the MXene heater melted completely within 30 min, with water temperature rising from −2 °C to 23 °C (Figure 16c). In contrast, control samples without heating remained fully frozen. The MXene film achieved an electrothermal deicing efficiency of 50.2%, outperforming the graphene film (41.4%), highlighting MXene’s potential for energy-efficient, low-voltage deicing systems.

### 5.4 Crude Oil Separation from Seawater

MXene-based Joule heating offers an effective approach for separating crude oil from seawater. Yang et al. developed a polyethylenimine (PEI)/polypyrrole­(Ppy)/MXene sponge by sequentially coating a melamine sponge with PEI, performing in situ pyrrole polymerization, and integrating MXene via combined electrostatic and covalent interactions (Figure 16d).^183^ This architecture enhanced interfacial stability and formed continuous heat conduction pathways for Joule heating. When a voltage of 5 V was applied, the sponge rapidly and uniformly heated from 20 °C to 100 °C, significantly reducing crude oil viscosity. Consequently, the sponge absorbed crude oil within 30 s while floating on the oil surface. To simulate practical oil spill scenarios, a vacuum-assisted oil recovery system was assembled using a peristaltic pump in combination with Joule heating. After 10 min of operation, the device recovered 9.58 g of crude oil, demonstrating strong potential for rapid and efficient oil spill remediation.

### 5.5 Thermal Actuation

MXene-based Joule heaters also show promise as low-voltage electrothermal actuators (ETAs). Sand et al. fabricated poly­(tetrafluoroethylene) (PTFE)/MXene/polyimide (PI) sandwich actuators via a simple “cutting and taping” technique.^184^ The actuation mechanism relies on the mismatch in thermal expansion between PTFE and PI layers. Upon voltage application, this thermal expansion difference causes the actuator to bend into an S-shaped profile. For more complex actuation, a four-finger soft gripper was constructed from U-shaped PTFE/MXene/PI actuators. At 6 V, the gripper transformed into a flower-like geometry, enabling adaptive grasping capabilities (Figure 16e). Additionally, the actuator was integrated into a smart window curtain system. With no voltage applied, the curtain remained closed, blocking light and electromagnetic interference. Whereas applying voltage triggered automatic retraction, allowing both light and electromagnetic radiation to pass. These demonstrations highlight the potential of MXene-enabled actuators for adaptive soft robotics and intelligent environmental control (Figure 16f).

## 6 Summary and Outlook

MXenes have emerged as versatile and highly promising materials for advanced thermal management technologies—including infrared heat shielding, thermal camouflage, photothermal conversion, and electrothermal heating—attributed to the combination of high electrical conductivity, tunable light absorption, low IR emissivity, and anisotropic thermal conductivity. This review has consolidated current understanding of their adjustable optical, thermal, electrical, and emissive characteristics, and analyzed their crucial roles in three primary domains: thermal camouflage, photothermal conversion, and electrothermal heaters.

The intrinsically low through-plane thermal conductivity and IR emissivity of MXenes make them ideal for thermal camouflage, insulation, and dynamic regulation of heat signatures. Their strong UV–vis-NIR absorption, combined with low thermal emission, enables efficient photothermal conversion for solar thermal harvesting, water purification, and biomedical therapies. Furthermore, high electrical conductivity paired with excellent thermal confinement ensures energy-efficient electrothermal heating for wearable heating systems, deicing devices, and thermal actuators. The compositional tunability, surface termination control, and diverse structural formats—films, coatings, aerogels, fibers, and composites—offer broad design flexibility for next-generation multifunctional thermal management systems.

Despite significant advances, several challenges remain before the full potential of MXenes can be realized. The structure–property relationships linking chemical composition, morphology, and thermal or spectral behavior are not yet fully elucidated. Addressing these knowledge gaps will require coordinated progress in fundamental understanding, materials design, and application engineering. The experimental characterization of thermal transport of MXenes needs more attention from researchers. Despite the limited available literature and often opposite opinions, the experimental values are lower than those of theoretical calculations. The high electronic conductivity of MXenes is supposed to promise quite a portion of thermal conductivity from the electron contribution; however, the violation of Wiedemann–Franz law with extremely low Lorentz number suggests a pathway to advanced thermal management. A great potential in controlling both phonon and electron scattering is expected to be utilized with an in-depth study of the restacking and layer-by-layer assembly of MXenes.

For thermal insulation and camouflage research, current MXene-based camouflage technologies focus primarily on midwave IR concealment, whereas real-world surveillance often involves multispectral detection—visible, NIR, radar, and laser. Future efforts should target adaptive multispectral camouflage by constructing hybrid architectures (e.g., layer-by-layer assembly), implementing compositional gradients, or integrating phase-change and thermochromic components to dynamically modulate emissivity and reflectance across broad spectral ranges. Enhancing thermal insulation through interlayer spacing control (via tailored terminations or intercalants) or termination engineering will further expand MXene use in extreme-temperature environments, particularly in aerospace and defense. However, with increasing availability and decreasing cost, one can imagine insulating industrial equipment, commercial buildings, and private housing using micrometer-thin MXene coatings. This may lead to a drastic decrease in energy consumption worldwide.

For photothermal energy conversion research, MXenes’ broadband solar absorption and low IR emissivity make them attractive for photothermal systems, but biomedical applications such as photothermal therapy and targeted drug delivery require precise absorption within biorelevant spectral windows (e.g., NIR). Beyond Ti_3_C_2_T_*x*_, composition engineering (transition metal selection, C/N stoichiometry) and surface chemistry tuning should be employed to align absorption peaks with medical laser wavelengths. Clinical translation will also depend on improving biocompatibility and biodegradability through polymer coatings like poly­(ethylene glycol) (PEG), poly­(vinylpyrrolidone) (PVP), biomolecule conjugation (proteins, peptides), or encapsulation in biodegradable carriers (hydrogels, liposomes). Halogen-free synthesis routes and biocompatible elemental compositions may further reduce safety concerns.

For electrothermal heating research, while MXenes are recognized for their exceptional Joule heating capability, the interplay of low out-of-plane thermal conductivity, high electrical conductivity, and low IR emissivity in determining heating efficiency is not yet quantitatively resolved. Developing predictive models to decouple these effects will be crucial for advancing electrothermal performance.

Furthermore, from material point of view, several advancements are needed for advancing thermal management performance. Most of all, to date, Ti_3_C_2_T_*x*_ has dominated research, leaving other compositions largely unexplored. The choice of transition metals, carbon/nitrogen stoichiometry, and surface terminations strongly influences phonon scattering, electronic transport, and optical properties. Integrated studies combining density functional theory (DFT), molecular dynamics simulations, and in situ spectroscopy are needed to establish universal structure–property relationships across the MXene family. Moreover, systematic studies correlating synthesis parameters with key IR-related properties—such as emissivity, solar absorptance, photothermal efficiency—remains scarce. This gap highlights the need for future research to establish quantitative synthesis–structure–property relationships enabling the rational design of MXenes with optimized IR characteristics and enhanced thermal management performance.

A major barrier to real-world deployment is MXenes’ susceptibility to oxidation, driven by surface dangling bonds and lattice defects, especially at edges and surfaces. Long-term stability can be improved through defect-minimized synthesis, defect-healing thermal treatment, and surface functionalization and passivation with small or polymeric molecules. Accelerated aging tests—thermal cycling, UV exposure, high-humidity storage—are essential for evaluating durability in aerospace and defense environments. Precursor-driven defect minimization strategies—such as controlling defect formation during the MAX phase synthesis—can effectively reduce structural imperfections and thereby enhance the intrinsic properties and oxidation stability of MXenes for real-world applications. Moreover, comprehensive studies of the effects of light and temperature exposure on MXene oxidation behavior, coupled with systematic studies of how oxidation influences thermal conductivity, absorptance, and emissivity, are essential for guiding the design of oxidation-resistant MXenes with superior long-term stability and performance.

The mechanical strength of pristine MXenes films and coatings depends on van der Waals or hydrogen interlayer bonding. Mechanical resilience can be further enhanced by embedding flakes in robust polymer matrices, forming covalent interlayer linkages, or applying cross-linking strategies, without compromising electrical or thermal performance. Such advancements are essential for wearable electronics, deployable sensing platforms, and military devices that face complex mechanical loading.

For thermal insulation and camouflage research, most reported studies have demonstrated passive thermal camouflage without tunability. By employing an in situ redox process in electrochemical cells, as illustrated in Figure 2f, the surface terminations of MXenes can be tuned in real time, enabling control over the emissivity. Such voltage-gated control of thermal properties allows MXenes to be utilized for active thermal camouflage with dynamic manipulation of infrared radiation. Realizing the full potential of MXene-based thermal management systems will also require sustained interdisciplinary collaboration across materials chemistry, device engineering, and computational modeling. Addressing the outlined challenges will accelerate the translation of MXenes into high-performance, durable, and multifunctional thermal solutions for defense, aerospace, healthcare, and next-generation consumer electronics.

## Vocablary

Infrared Thermal Management: refers to the control and manipulation of heat radiation in the infrared spectrum to regulate temperature, governing how materials absorb, reflect, and emit infrared energy for thermal regulation, energy saving, and camouflage
Infrared Emissivity: measures how effectively a surface emits thermal radiation relative to a blackbody, depending on electronic structure and surface roughness, and determines radiative heat loss and infrared visibility
Thermal Camouflage: is the strategy to conceal an object’s infrared signature by controlling surface temperature or emissivity, minimizing heat contrast with the environment to avoid IR detection
Electrothermal: describes heat generation or control through electrical energy, physically based on Joule heating — the conversion of electrical current into thermal energy due to material resistance
Photothermal: involves the conversion of absorbed light, typically visible or infrared, into heat through nonradiative relaxation of photoexcited electrons, useful in solar energy, therapy, and sensors
